# Disparities in correlating microstructural to nanostructural preservation of dinosaur femoral bones

**DOI:** 10.1038/srep45562

**Published:** 2017-03-30

**Authors:** Jung-Kyun Kim, Yong-Eun Kwon, Sang-Gil Lee, Ji-Hyun Lee, Jin-Gyu Kim, Min Huh, Eunji Lee, Youn-Joong Kim

**Affiliations:** 1 Graduate School of Analytical Science and Technology (GRAST), Chungnam National University, 99 Daehak-ro, Yuseong-gu, Daejeon, 34134, South Korea; 2 Center for Electron Microscopic Research, Korea Basic Science Institute (KBSI), 169-148 Gwahak-ro, Yuseong-gu, Daejeon, 34133, South Korea; 3 Korea Dinosaur Research Center & Department of Earth Systems and Environmental Sciences, Chonnam National University, 77 Yongbong-ro, Buk-gu, Gwangju, 61186, South Korea

## Abstract

Osteohistological researches on dinosaurs are well documented, but descriptions of direct correlations between the bone microstructure and corresponding nanostructure are currently lacking. By applying correlative microscopy, we aimed to verify that well-preserved osteohistological features correlate with pristine fossil bone nanostructures from the femoral bones of *Koreanosaurus boseongensis*. The quality of nanostructural preservation was evaluated based on the preferred orientation level of apatite crystals obtained from selected area electron diffraction (SAED) patterns and by measuring the “arcs” from the {100} and {002} diffraction rings. Unlike our expectations, our results revealed that well-preserved microstructures do not guarantee pristine nanostructures and vice versa. Structural preservation of bone from macro- to nanoscale primarily depends on original bioapatite density, and subsequent taphonomical factors such as effects from burial, pressure, influx of external elements and the rate of diagenetic alteration of apatite crystals. Our findings suggest that the efficient application of SAED analysis opens the opportunity for comprehensive nanostructural investigations of bone.

Many recent studies on the osteohistology of dinosaurs have yielded invaluable insights on their paleobiological aspects, especially growth, from an evolutionary perspective along with advances in analytical and interpretation methods^1^,^2^,^3^. Using electron microscopy (EM), structural investigation of a dinosaur bone is achievable at the nanoscale, further advancing the understanding of fossil bone structure and its taphonomical and paleobiological implications^4^,^5^,^6^,^7^,^8^,^9^,^10^. With the rapid advancement of three-dimensional (3-D) microtomographic techniques, intrinsic 3-D visualization of an interior dinosaur bone microstructure can be achieved without physical destruction^11^ and is being widely applied. However, there is a notable lack of studies focused on direct correlation between dinosaur osteohistology and corresponding bone nanostructure although the preferred orientation of apatite crystals in dinosaur bones indicating nanostructural preservation has been documented^4^,^8^,^9^,^12^. While a method of investigating extant bone nanostructures in three dimensions using small-angle scattering tensor tomography has been recently developed^13^, in fossilized bones, several mineral phases originating from the depositional environment and its effects must be taken into account^5^,^7^,^14^,^15^. The continuous advances in EM combined with a variety of analytical techniques have greatly enhanced the understanding of general bone nanostructure and chemistry^16^,^17^,^18^,^19^,^20^,^21^,^22^,^23^,^24^,^25^,^26^,^27^. Such studies applied in fossils have revealed the supposed identity of organic phases in 75-million-year-old dinosaur bones^28^,^29^.

Transmission electron microscopy (TEM) is ideal for specific and direct nanostructural investigations of fossil bone. However, the main issue of TEM investigation is that the individual data only represents an extremely small portion of the fossil, thus, a large number of data is required for generalization. In this respect, correlative microscopy is an important method of compensating for the shortcomings of TEM analysis as spatial, structural and chemical information can be maintained in differing scales^7^. Prior to nanoscale investigations by TEM, correlative optical microscopy (OM) data on fossil bone microstructure and macroscopic features are essential for assessing the paleobiological and taphonomical implications that may have affected structural properties^2^,^3^. Scanning electron microscopy (SEM) is capable of evaluating the detailed fossil bone surface morphology and specific structures from low to high magnification in a 3-D perspective. Chemical analysis on a relatively wide scale can also be achieved. Therefore, SEM analysis provides an effective middle ground between OM and TEM studies^7^. For preparation of TEM samples directly from the optical thin sections without the loss of spatial information, focused ion beam (FIB) milling proved to be very efficient at obtaining samples in different orientations, and produced less dissolution compared to ultramicrotomy^30^. For nanostructure analysis, we have obtained SAED (selected area electron diffraction) patterns of apatite crystals without using a beam stopper, since important crystallographic information for the large d-spacing values are obscured when the beam stopper is present. From the SAED patterns, the “arcs” of the {100} and {002} diffraction rings were measured to evaluate the level of the preferred orientation of apatite crystals (Supplementary Fig. S1).

The main assumption we had on fossil bone structure was that a well-preserved microstructure is maintained through a foundation of well-preserved nanostructure. Here, we describe correlative structural investigations of the right (associated with KDRC-BB1) and left (associated with KDRC-BB3) femora of *Koreanosaurus boseongensis*^31^ from the Late Cretaceous dinosaur nesting sites in Boseong County^31^,^32^,^33^,^34^,^35^,^36^, South Korea to elucidate such assumptions (see Supplementary Table S1 for specimen details). *Koreanosaurus* was a small basal ornithopod that may have been fossorial based on the context of its discovery, suggested enlarged forelimb elements and pectoral girdle^31^, and comparisons with a fossorial ornithopod *Oryctodromeus cubicularis* from Montana, USA^37^,^38^,^39^. We specifically selected both femora as our research specimens owing to their well-preserved state at the macroscale (Fig. 1a,b), information on precise excavation sites (Supplementary Fig. S2) and initial state of discovery (Supplementary Table S1). Fossilized femoral bones also typically display well-preserved osteohistological features owing to the compact nature of the bone cortex^1^,^2^,^3^,^40^,^41^. Although the right femur does not ideally represent microstructural preservation, it is notably better preserved than the left femur in microscale as it has more pronounced osteohistological features and the extent of damage of the bone wall caused by externally originating phases is less prevalent. Therefore, we have prioritized it in our correlative structural analysis. While our primary aim of the study was verifying the correlation between the preservation of microstructure and nanostructure of fossil bone based on our specimens, we have also focused on investigating how biological and taphonomical factors may affect bone structure on varying levels, and establishing efficient TEM methods for in-depth analysis of bone nanostructure for further applications.

## Results

### Microstructural analysis

**Figure 1:**
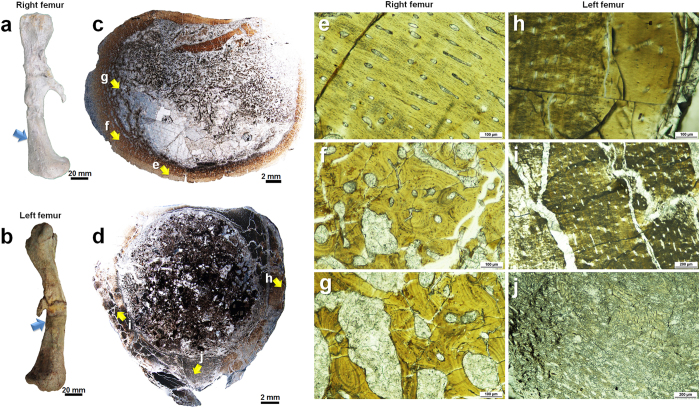
Gross morphology and selected osteohistological features of both femora. (**a**,**b**) Medial view of both femora. The arrows indicate sectioned locations. (**c**,**d**) Overall composite micrographs of optical thin sections from both femora. (**e**–**j**) Magnified optical micrographs obtained from the regions marked by yellow arrows in (**c**,**d**). The osteohistological features of the right femur are preserved at a relatively higher degree in microscale. Detailed optical micrographs of both femora are provided in Supplementary Fig. S3.

Overall composite micrographs of the optical thin sections of both femora (Fig. 1c,d) and selected microstructural features (Fig. 1e–j) are highlighted in Fig. 1. From the right femur, the overall bone wall surrounding the medullary cavity is thick and richly vascularized, and no endosteally derived tissue was evident. The bone tissue of the outermost bone wall appears to be comprised of parallel-fibred bone with woven bone content and has high vascularity (Fig. 1e and Supplementary Fig. S3a,b). The middle cortical layer, which has a wide distribution (Fig. 1c), mainly comprises woven bone matrix with sporadic to relatively populous distribution of secondary osteons, and resorption spaces can be observed (Fig. 1f and Supplementary Fig. S3c–e). Certain features indicate that this particular region still retained the bone microstructure formed during early ontogeny^1^,^2^,^39^,^40^,^41^,^42^,^43^. The size and distribution of resorption spaces increases towards the medullary cavity, making the bone appear osteoporotic starting from the middle cortical layer. This may be attributed to age or could represent reproductive strategies similar to that of crocodilians^44^,^45^, and may also be due to the wider distribution of cancellous bone closer to the metaphyseal region from where our sections were also obtained (Fig. 1g and Supplementary Fig. S3f,g). However, especially when focused on the middle cortical layer, the exact nature of such features is currently unclear and requires further study. The majority of the “struts” previously forming the cancellous bone have broken off, and the fragments shifted toward the posterior region, leaving a large void filled with calcite at the anterior region of the medullary cavity (Fig. 1c). The lack of an external fundamental system (EFS) indicates that this particular individual may have not reached full skeletal maturity^1^,^2^. Other growth features such as lines of arrested growth (LAGs) were not observed. Therefore, it is likely that this individual has not reached maximum skeletal size.

The preservation state of osteohistological features of the left femur is considerably poor although the vascularization pattern is discernible, and the overall bone wall is very thick (Fig. 1d). The outermost bone wall is poorly vascularized and appears to be very compact (Fig. 1h and Supplementary Fig. S3h,i). The widely distributed inner bone wall region is richly vascularized although precise bone tissue type is uncertain owing to poor preservation (Fig. 1i and Supplementary Fig. S3j–l). The anterior inner bone wall has bone matrices with low density, thus the interior of the keeled region is highly porous (Fig. 1j and Supplementary Fig. S3m,n). The hindering of osteohistological features was probably caused by extensive pressure and calcite intrusion (Fig. 1d,i and Supplementary Fig. S3j,k).

### Comparative structural analysis

**Figure 2:**
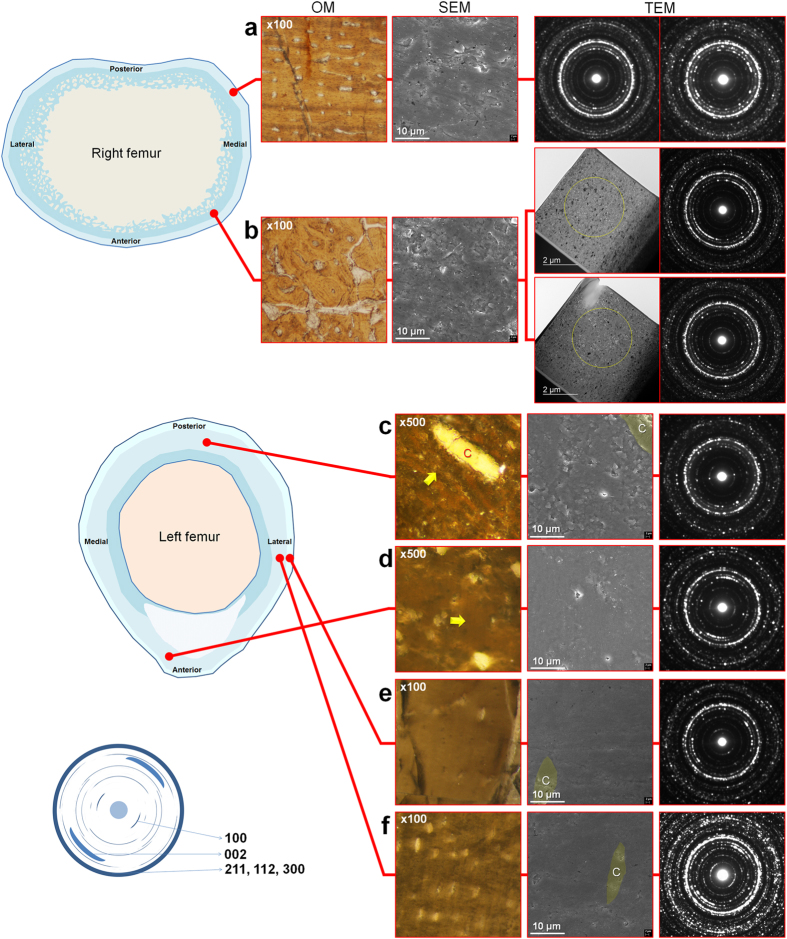
Comparative OM-SEM-TEM structural analyses of both femora. The illustrated overall cross-sections represent estimated and simplified images of both femora being in pristine condition. The illustrated SAED pattern of fossil bone apatite represents a high degree of preferred orientation of apatite crystals. The SEM micrographs represent areas adjacent to the FIB-milled regions with consistent surface morphology and preservation features from the exact milled spots (see Supplementary Fig. S4 for detailed SEM micrographs). The TEM data reveals the quality of nanostructural preservation based on the SAED patterns of apatite crystals. (**a**,**b**) Right femur. Although osteohistological features are well-preserved throughout the sample as shown in the representative optical micrographs, the general apatite density and its arrangement within the bone matrix appeared to be uneven in EM (SEM + TEM) data. (**a**) Outermost bone wall comprised of parallel-fibred bone. (**b**) Middle bone wall. Yellow circles in TEM micrographs indicate the designated areas for obtaining SAED patterns. (**c**–**f**) Left femur. The OM-SEM-TEM data on structure was relatively consistent. Yellow arrows in OM micrographs from (**c**,**d**) indicate the exact designated regions viewed in SEM. C = calcite. (**c**) Inner bone wall region with small-scale calcite intrusion. (**d**) High preservation level of nanostructure in compact bone areas with poorly preserved microstructure. (**e**) Relatively intact outermost bone wall, and (**f**) relatively intact inner bone wall displaying high level of nanostructural preservation.

An overall comparative structural correlation using OM, SEM, and TEM of both femora is provided in Fig. 2. Representative regions of bone tissue were selected from both femora for direct comparison. From the left femur, we have also focused on regions based on contrasting levels of microstructural preservation for comparative EM analysis. For TEM investigations, we prepared FIB-milled samples directly from the designated regions within the optical thin sections of both femora (see *Methods* for details). We performed thorough SEM-energy dispersive spectroscopy (EDS) mapping analysis throughout the bone walls of both femora and low- to high-magnification SEM imaging of the bone surface focused on areas primarily comprising apatite (Supplementary Fig. S4) prior to and post FIB-milling. As both femora optical thin section samples were prepared in identical conditions, we can exclude the possibility that differing surface polishing levels affected the SEM image quality. The parallel-fibred bone from the right femur exhibited a less rough surface morphology (Fig. 2a and Supplementary Fig. S4a) as the region may have had a relatively even distribution of apatite density. The surface morphology of the inner bone wall (Fig. 2b and Supplementary Fig. S4b) appeared to be very rough because apatite density levels differed throughout the region. Although not readily discernible from surface morphology, SAED patterns inform that the preferred orientation of apatite crystals is variable based on TEM samples obtained from the same region (Fig. 2b), indicating the differing levels of localized nanostructural preservation. Thus, the bone matrix of the right femur has a mixed distribution of well- to poorly preserved nanostructures throughout the bone wall, and these features cannot be discerned without SAED analysis. In contrast, for the left femur, the SEM-imaged surface morphology was consistent with the OM and TEM data (Fig. 2c–f). In regions with relatively intact microstructures, the apatite density and arrangement were consistent, resulting in smooth surface morphology even at high magnifications (Supplementary Fig. S4c). On the other hand, bone matrix regions affected by calcite intrusion displayed varying apatite density levels and demonstrated the overall disrupted structure, revealing the poor structural preservation from the microscale to the nanoscale (Fig. 2c, Supplementary Fig. S4d). Although the surface morphology revealed by SEM imaging is not a definitive indication of how the nanostructure is preserved, it can still be used as a valuable reference for predicting the potential outcome from TEM nanostructural analysis, thus allowing us to select desired regions for FIB milling, as exhibited in the left femur.

### In-depth nanostructural analysis

From a biological perspective, the initial nanostructure of femoral bone is linked with its arrangement of bioapatite crystals which heavily depends on the collagen type 1 framework^16^,^18^,^19^,^20^,^21^,^24^. While bioapatite crystals tend to have a more elongated and sometimes needle-like shape^16^,^18^,^19^,^20^,^21^,^24^,^26^, apatite crystals from fossil bones typically have platelet-like shapes with a greater short-axis length^5^,^6^,^7^,^8^ which is also shown in our study (Supplementary Fig. S5). It has been demonstrated that heat treatment of bioapatite causes an increase in apatite crystallinity^26^ and along with pressure and the influx and interaction of elements from the depositional environment, diagenetic alteration of apatite occurs^46^,^47^,^48^,^49^. The increased crystallinity and dimension of diagenetically altered apatite in fossils may directly contribute to the preservation of fossil bone nanostructure as the open spaces left by decomposed collagen become reduced^48^,^49^.

**Figure 3:**
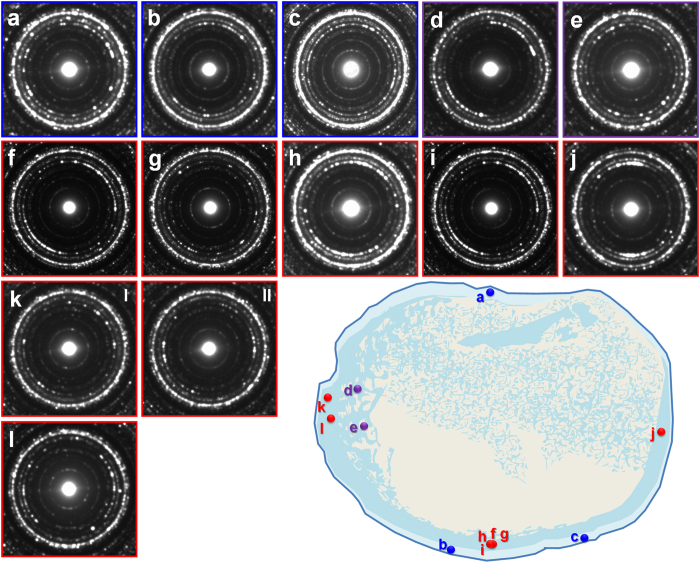
SAED patterns of the right femur from cross-FIB-milled samples. The nanostructure is generally preserved in varying degrees from all regions. (**a**–**c**) Outermost bone wall comprised of parallel-fibred bone (**c** is specifically from an upper thin section from the marked region). (**d**,**e**) Innermost bone wall with cancellous bone. (**f**–**l**) Middle bone wall. Corresponding OM, SEM, and TEM micrographs in Supplementary Fig. S8.

**Figure 4:**
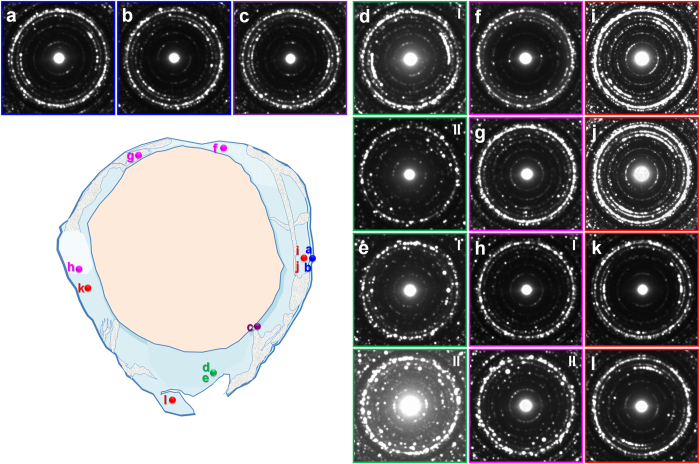
SAED patterns of the left femur from cross-FIB-milled samples. Regions with intact bone tissue had well-preserved nanostructure. Disrupted regions (mainly by calcite intrusion) eventually lost their structural integrity from the microscale to the nanoscale. (**a**,**b**) Outermost bone wall. (**c**) Innermost bone wall. (**d**,**e**) Anterior inner porous region. (**f**–**h**) Disrupted inner bone wall. (**i**-**l**) Intact inner bone wall. Corresponding OM and TEM micrographs in Supplementary Fig. S9.

The indexing of the SAED patterns from the fossilized femora (Supplementary Fig. S6) was primarily based on the fluorapatite data provided by Hughes *et al*.^50^. The SAED data obtained from an extant mouse femur (Supplementary Fig. S7) was used to compare the degree of the fossil bone retaining its original nanostructure. The overall results from SAED pattern analysis from cross-FIB-milled samples are shown in Figs 3 and 4; the results represent the orientation of apatite crystals aligned along the long axis of both femora. The exact milled locations and corresponding TEM micrographs are provided in Supplementary Figures S8 and S9. As the SAED patterns were obtained without the beam stopper utilizing the side charge-coupled device (CCD) camera (see *Methods*), we were able to evaluate the level of preferred orientation of apatite crystals based on the patterns and intensity of distinct “arc” rings of both the {100} and {002} diffractions, which are perpendicularly arranged. The levels of preferred orientation of apatite crystals were evaluated based on the shape of the {002} “arc”, referring to that of the {100} “arc” (Table 1, Supplementary Table S2).

As shown in Fig. 3, the nanostructure of the right femur was generally uneven (Fig. 2a,b and Supplementary Fig. S8). Most of the acquired SAED patterns did not show a high level of preferred orientation. Based on our results, we were able to confirm that unevenly preserved nanostructures did not notably affect the appearance of osteohistological features. The left femur displayed much better preservation of nanostructure in regions wherein the microstructure was relatively intact (Fig. 4a,b,i–k and Supplementary Fig. S9a,b,i–k). Regions affected by small-scale (Fig. 4f and Supplementary Fig. S9f) and large-scale calcite intrusion (Fig. 4g and Supplementary Fig. S9g) show the hindering of both the microstructure and nanostructure. Therefore, a severe collapse of nanostructure can be linked with disrupted microstructural features. From the anterior inner porous region rich in clay phases, the sample with sufficient amount of apatite crystals (Fig. 4dI, Supplementary Fig. S9dI) had a strong degree of preferred orientation. With an exception of a few samples, all plane-FIB-milled samples (perpendicular to the long axis of the femur) from both femora exhibited a generally random orientation of apatite crystals (Supplementary Fig. S10). The very weak intensity of the {002} plane in all the left femur samples further indicates that the apatite crystals are aligned along the long axis of the femur.

### Calcite and clay distribution in both femora

**Figure 5:**
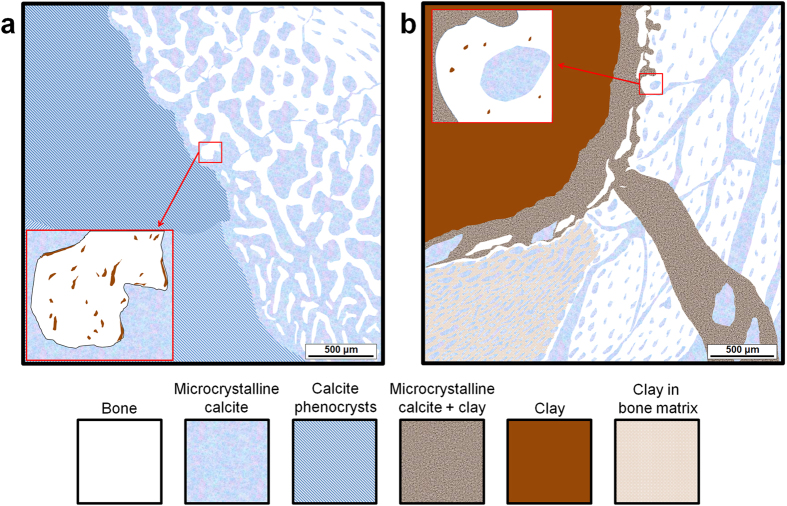
Simplified illustration of calcite and clay distribution in both femora. The illustration represents the main distribution trend of these externally originating phases revealed through cross-polarized OM imaging, SEM-EDS and EPMA-WDS mapping. Also, note that the illustration is not from exactly matching specific regions from both femora. The insets represent the distribution of clay occupying miniscule pores within the bone. The distribution of these pores decreases towards the outer bone wall. (**a**) Right femur. Large calcite phenocrysts are present within the medullary cavity in areas lacking bone fragments. (**b**) Left femur. The highly destructive nature of intrusive calcite is shown, and the mixed distribution of clay and calcite is also a notable feature exclusive to the left femur. The anterior inner porous region has a wide distribution of clay within the bone matrix.

The distribution of calcite and clay in both femora was investigated due to their abundance from the depositional environment and their supposed role in taphonomy^31^,^32^,^33^,^34^,^35^,^36^,^51^, and Fig. 5 illustrates the overall distribution trend of these phases. X-ray diffraction (XRD) analysis from both femora revealed that calcite was the major constituting phase (Supplementary Fig. S11). The formation of clusters of calcite microcrystals within both femora indicates that the specimens went under dehydration and pressure^52^ (Supplementary Figs S12 and S13). The well-preserved nature of osteohistological features from the right femur, even in broken off bone walls (Supplementary Fig. S12d) implies that calcite has not initially affected the bone when it was in a fresh state. Besides the fracturing of cancellous bone, the destructive process of calcite was relatively minimal, and may have even aided the preservation of bone, especially once the surrounding calcite nodule was fully formed^31^,^47^,^51^. In the left femur, post-burial pressure combined with the influx and crystallization of solubilized calcite has resulted in the fracturing of the bone wall in varying scales (Supplementary Fig. S13). This process seemed to have occurred while the bone was still in a fresh state (Supplementary Fig. S13a,b) up to full lithification (Supplementary Fig. S13c–f). The thick bone wall with the poorly vascularized outermost layer and the surrounding mud may have minimized exit points for the excess calcite that has built up within the bone, and ended up as intrusive calcite distributed throughout the bone wall. The crystallization pattern of calcite (Supplementary Fig. S13a–e) is notably less uniform compared to the right femur.

SEM-EDS and electron probe microanalyzer with wavelength dispersive spectroscopy (EPMA-WDS) analyses were employed to identify specific clay phases based on their chemistry^53^,^54^ and to evaluate their distribution in both femora. Although clay phases were not detected by XRD from the right femur, EPMA-WDS analysis revealed that illite was sparsely distributed in miniscule pores (Supplementary Fig. S14a). The distribution of clay phases in the left femur was more complex, although the clay filling the miniscule pores in dense bone matrix regions was primarily illite, as in the right femur. In the anterior inner porous region, only the lower left portion displayed high illite concentration (Supplementary Fig. S14b), and the region was widely composed of multiple clay phases (Supplementary Fig. S15). The significance of clay phases distributed in highly porous bone matrices is their suggested role of aiding the preservation of bone (Fig. 4d and Supplementary Fig. S9d).

## Discussion

Based on the right femur, unlike what we have initially expected, there was a correlational disparity between the microstructural and nanostructural preservation. While the left femur showed more consistent results, it has also demonstrated that high nanostructural preservation levels can be maintained from regions with subpar preservation level of microstructures (Fig. 2d and Supplementary Fig. S9c,d,l). Such structural features were resulted from how the specimens met their demises, how fossilization occurred, and the density level of the original bone apatite^31^,^33^,^46^,^47^,^48^,^49^,^51^,^55^. Table 2 summarizes our results and interpretation of structural preservation. For comprehensive nanostructural analysis of fossil bone by TEM, the significance and necessity of obtaining proper SAED patterns is emphasized. Evaluating the preferred orientation of apatite crystals primarily based on TEM micrographs alone is unreliable, and based on our results (Figs 2, 3, 4 and Supplementary Figs S8 and S9), we suggest that SAED patterns provide a much simpler and clearer indication of apatite arrangement in specific areas compared to other known methods for investigating the ultrastructure of bone^56^. We were able to perform a more thorough and reliable analysis of SAED patterns removing the beam stopper, since apatite diffraction rings from planes with large d-spacing values, especially the {100} planes, do not usually overlap with diffraction rings of other phases that may be present.

The initial process the specimens experienced after death holds the key of how the fossilized remains ended up^55^. The individual associated with the right femur appears to have been on the surface for prolong periods of time after its death, which is evidenced by the disarticulated nature of KDRC-BB1, heavy surface erosion, and the lack of clay in the medullary cavity. The well-preserved microstructure is attributed to minimal effects from physically destructive processes during initial fossilization. Due to the slower rate of diagenetic alteration of apatite crystals, after full decomposition of the organic phases, the empty spaces between apatite crystals may have caused collapse of the original arrangement, albeit at a very small scale with minimal effect on the overall microstructure owing to the dense femoral bone matrices. This may explain the disparity in correlation of the microstructure and nanostructure. Presumably as a result of seasonal flooding^33^,^36^, the right femur experienced subsequent burial by mud. Clay precipitation in bone likely originated from subsequent burials. On the other hand, the individual associated with the left femur experienced rapid burial, and burial itself may have been the cause of its demise^31^. The articulated nature^55^ of KDRC-BB3, the extensive pressure that was apparent in most elements of the skeleton and clay filling the medullary cavity provide direct evidence of rapid burial taking place. Although extensive pressure may have removed osteohistological details of the left femur, it may also have contributed to preserving bone nanostructure by reducing the spaces between apatite, resulting in higher density. Thus, the originally dense bone wall, applied pressure, faster rate of apatite diagenesis all seemed to have contributed in certain regions maintaining excellent level of bone nanostructure, which agrees with the model of nanostructural bone preservation provided by Trueman and Tuross^48^. Nanostructural preservation of highly porous bone with low apatite density can be maintained with the precipitation of clay phases and its subsequent diagenesis in miniscule pores within the bone matrix. The platy morphology of clay^53^,^54^ seems to make it compatible with constrained spaces between groups of apatite crystals. This process significantly increases the density of the affected area, thus contributes to maintaining the structural integrity of bone. This feature is observed in the highly porous anterior inner portion of the left femur (Fig. 4dI). However, as the overall bone matrix of the right femur sample was dense and the femur itself was encased in a calcite nodule, the effect of clay on its nanostructural preservation was probably negligible.

Besides taphonomical factors, it is also possible that rapidly formed woven bone tissue had higher proportion of random apatite orientation which might additionally explain why the right femur had less organized apatite arrangement. However, without direct comparative and comprehensive TEM data from woven bone tissues obtained from extant femoral bone at varying ontogenetic stages, it is difficult to confirm the effect of developmental factors on nanostructural features. On the other hand, SAED analysis on human fetal woven bone tissue obtained from femoral bones did display a certain level of preferred orientation of apatite crystals^24^. Our work on extant femoral bones (not limited to mice) from TEM samples parallel to the long axis of the femur also consistently resulted in SAED patterns displaying preferred orientation of apatite crystals, although specific bone tissue type was not primarily considered. Collagen fibrils that are not well-organized based on TEM micrographs may still result in SAED patterns with highly organized apatite orientation because the apatite crystals within the fibrils are facing a specific direction (Supplementary Fig. S7).

In summary, the processes involved in the nanostructural preservation of fossil bone should be considered at a different perspective from bioapatite which follows the collagen framework^16^,^17^,^18^,^19^,^20^,^21^, and biomimetic apatite which tend to grow in the c-axis direction^57^. Based on our studies, we suggest that the nanostructure from dense bone regions is primarily maintained from the increase in size and crystallinity of diagenetically altered apatite crystals^48^,^49^. The rate of diagenetic alteration is also an important factor, and the rate increases if the bone is in a buried state. We suggest that pressure may be beneficial to a certain extent as it reduces open spaces between apatite crystals, as demonstrated in the left femur. In regions with low bone density, the association of clay phases helps increase the overall density, thus allowing the bone to maintain its structural integrity. For comprehensive nanostructural evaluation of bone, SAED analysis of the region of interest is a simple, yet crucial process, and we highly recommend its application for research on general bone biology, forensic science, anthropology, and vertebrate paleontology.

## Methods

### Sample collection and preparation

The right femur (KDRC-BB1) and the left femur (KDRC-BB3) were selected based on their relative completeness in macroscale, and information on precise location (Supplementary Fig. S1) and state of discovery. Specimen details are provided in Supplementary Table S1. We initially aimed to obtain sections from the mid-diaphyseal region, but as that region of the right femur was not available owing to previous studies, sections were obtained from the lower diaphysis/upper distal metaphysis instead. Sections from the left femur were directly obtained from the desired mid-diaphyseal region. The femora were initially cut using a stone-cutting saw housed at Chonnam National University. The sections were subsequently embedded in polyester resin and cured at room temperature for approximately 48 h. The embedded sections were cut using a diamond saw (Buhler Precision Saw) to prepare optical thin sections. Although two optical thin sections were prepared from each individual femur, only the lower section of the right femur and upper section of the left femur were primarily used for correlative microscopy. In case of the left femur, the lower section was used only for additional OM and XRD data.

### OM analysis

After polishing and mounting on a slide glass, the exposed side was initially ground in a wedge shape in anterior view to evaluate the ideal thickness for osteohistological investigations. For the right femur, the microstructure was best displayed at a sample thickness of approximately 40–60 μm. The bone textures of the left femur were visible at a sample thickness of approximately 70 μm, but the desired thickness was difficult to discern owing to its poor microstructural preservation. Initial observations on the prepared optical thin sections were conducted using a stereoscopic zoom microscope (Nikon SMZ 1500). Detailed observations of osteohistological features were performed using polarizing optical microscopes (Nikon Eclipse E600 Pol; Carl Zeiss Axiophot).

### XRD analysis

Initial XRD measurements were directly conducted on optical thin sections of both femora using a high-resolution XRD instrument (Bruker D8 Discover). The 2θ range was from 5° to 100° in steps of 0.02°; the duration of each step was 76.8 s. Peaks were assigned with the software DIFFRAC.EVA Ver.3 (Bruker). Subsequent measurements for data clarification were performed using an X’Pert-PRO materials research diffractometer (PANalytical). The 2θ range was from 5° to 90° in steps of 0.013°; the duration of each step was 31 s. Peaks were manually assigned primarily based on initial data from the Bruker instrument.

### SEM imaging and EDS analyses

Optical thin sections of both femora were coated with carbon with a coating thickness of approximately 20 nm. SEM imaging and SEM-EDS analyses were conducted based on the data obtained from OM observations and XRD analysis. Initial SEM observation was performed with an environmental SEM (Carl Zeiss LEO 1475VP) operating at 20 kV equipped with a backscattered electron (BSE) detector and an EDS detector (Vantage LN2 type). BSE imaging was performed to differentiate phases in SEM images of the sample, and EDS point scan analysis with spot sizes of 10–50 μm was conducted to clarify the identity of phases with different contrasts. Further, EDS point scan analysis was utilized for identifying the chemical composition on surfaces with different textures. To obtain high-magnification SEM images ( >10 k magnification) and EDS maps, a field emission SEM (FE-SEM, Carl Zeiss Merlin) operating at 0.02–30 kV equipped with an EDS detector (Bruker XFlash 6160) was employed. SEM micrographs were primarily obtained using a detector collecting secondary electron 1 (SE1/InLens) signals as it was capable of producing high resolution images showing individual apatite grains despite its lower level of contrast compared to the SE2 signals. SEM micrographs taken post TEM sampling were from specific regions adjacent and with bone tissues and preservation features consistent with the FIB-milled areas. EDS mapping was conducted for F, Na, Mg, Al, Si, P, K, Ca, and Fe. Data were analyzed with the software Espirit 1.9.4 (Bruker) and its subsequent updated versions up to 2.0.3.

### EPMA analysis

We used an EPMA instrument (Schmadzu Model 1610) with a tungsten gun operating at 15 kV–30 kV (20 kV mainly used) with a maximum current of 100 μA and equipped with four WDS detectors. We selected the following specific elements for quantitative chemical analysis and elemental distribution mapping using the built-in software program (Schmadzu): Na, Mg, Al, Si, P, K, Ca, and Fe. We primarily focused on mapping regions with high clay content in both femora based on the data from SEM-EDS mapping to verify specific clay phases.

### TEM sample preparation with FIB

TEM samples from both femora were directly prepared from the optical thin sections via a FIB miller (FEI Quanta 3D FEG) equipped with a FE gun and gallium ion beam operating at 0.5–30 kV (20 kV was mainly used; 2 kV used for polishing), with a beam current range of 1.5 pA–65 nA and a spot size range of 1.0–9.0. Specific regions of interest were marked after EDS spot scan analysis (Apollo X, Genesis Spectrum version 6.41). Regions for milling were specifically selected after SEM analysis based on bone density, the preservation level of osteohistological features, and the inclusion of clay phases in the highly porous bone region from the left femur. All the milled regions exceeded at least 10 μm in thickness from the optical thin sections in order to obtain sufficient amount of apatite crystals. Both cross and plane samples were prepared and loaded on specialized TEM grids (Omniprobe Lift-Out Grids). The dimensions of cross-FIB-milled samples (parallel to the long axis of the femur) were 10 × 5 μm, with 5 × 5 μm viewable area and 80 nm in thickness. Plane-FIB-milled samples (perpendicular to the long axis of the femur) were 10 × 3 μm, with 5 × 2~3 μm viewable area and 80 nm in thickness.

### TEM analysis

A field emission TEM (FE-TEM, JEOL JEM-2100F, operating at 200 kV) and a monochromated field emission energy-filtering TEM (FE-EFTEM, Carl Zeiss Libra MC, operating at 60–200 kV; 200 kV used in this study) were employed to obtain TEM micrographs, SAED patterns, high-resolution TEM (HRTEM) images, and EDS analysis from FIB-milled samples. SAED pattern acquisition and EDS analysis (Oxford X-Max 80T, Aztec version v2.2 SP2) were conducted using the FE-EFTEM. TEM-EDS analysis was primarily carried out to clarify the identification of clay phases from the left femur. The side CCD camera (Gatan Orius SC200D) specifications are 2048 × 2048 sensor size, 7.4 μm pixel size, and is capable of obtaining SAED patterns without a beam stopper. Although there were a wide range of selected area (SA) aperture sizes, we have used two specific sizes in this study. The largest SA aperture which has a diameter size of 3.2 μm (actual aperture size: 120 μm) on the TEM micrograph was primarily used. After testing the efficacy of the largest SA aperture on several FIB-milled samples, we have utilized it because it adequately displayed the general orientation trend of the apatite crystals without notable intensity fluctuations in diffraction rings. Moreover, we were able to obtain ED patterns using consistent parameters for each sample when we applied the SA aperture. The smaller aperture which has a diameter size of 1.6 μm (actual aperture size: 60 μm) on the TEM micrograph was mainly employed on samples containing other phases. Instances of particular samples with more than 1 SAED data used: Fig. 3k (the level of preferred orientation varied slightly from different areas), Fig. 4d,e (due to multiple phases and patchy distribution of apatite crystals), Fig. 4h (for demonstrating that while the orientation trend is consistent from each pattern, the intensity of the diffraction rings vary by using SA apertures with different sizes).

### SAED pattern analysis

The d-spacing values of SAED patterns were measured manually and with the software Digital Micrograph version 1.72.53 (Gatan). SAED patterns were processed and overlapped with Photoshop CS3 (Adobe) to verify that the patterns were consistent from different samples, and also to aid with the indexing (Supplementary Fig. S16). For evaluating the level of preferred orientation of apatite crystals based on the {100} and {002} diffraction rings, we have applied the following calculation:


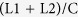


L is the arc length from the diffraction rings, and C is the circumference length. As “arcs” appear in each side and may slightly differ in length, both L values from each diffraction ring were acquired (Supplementary Fig. S1). The level of preferred orientation was designated primarily based on the ratio value from the {002} diffraction rings, and the {100} diffraction ring ratio values were used as a reference (Supplementary Table S2). Intensity levels of the diffraction rings (measured and evaluated with Digital Micrograph) were also taken into account, but were not directly applied due to reproducibility issues.

## Additional Information

**How to cite this article:** Kim, J.-K. *et al*. Disparities in correlating microstructural to nanostructural preservation of dinosaur femoral bones. *Sci. Rep.* **7**, 45562; doi: 10.1038/srep45562 (2017).

**Publisher's note:** Springer Nature remains neutral with regard to jurisdictional claims in published maps and institutional affiliations.

## Supplementary Material

Supplementary Information

## Figures and Tables

**Table 1:** Preferred orientation of apatite crystals in both femora.

Microstructure		Right femur	Corresponding figure(s)	Left femur	Corresponding figure(s)
Intact regions	Outer bone wall	Weak	3b	Strong	4a; 4b
		None	3a; 3c		
	Inner bone wall	Strong	3j	Strong	4i; 4k; 4l
		Moderate	3h; 3i	Moderate	4c; 4j
		Weak	3f; 3kII; 3l		
		None	3g; 3kI		
Unique regions	Innermost bone wall with cancellous bone	Strong	3d	—	—
		None	3e	—	—
	Disrupted regions	—	—	Weak	4f
		—	—	None	4g; 4hI; 4hII
	Anterior inner porous region	—	—	Strong	4dI
		—	—	None	4dII; 4eI; 4eII

**Table 2:** Comparative analysis of fossil bone preservation from the macroscale to the nanoscale.

	Right Femur	Left Femur
Gross Morphology	Overall shape preserved, proximal region shows heavy erosion.	Well-preserved, surface of distal end show signs of erosion.
Microstructure	- Relatively well-preserved.	- Considerably hindered.
	- Vascularization pattern intact.	- Vascularization pattern intact.
	- Fragmentation of cancellous bone and certain areas of the bone wall.	- Bone tissue type of highly porous region not discernible.
Apatite Density and Distribution	- High and generally uneven.	- High and generally even in intact bone regions.
	- Outer bone wall distribution is relatively more even.	- Only the anterior inner porous region has low density.
Preferred Orientation of Apatite	Varying degrees in every sampled region.	- High degree in intact bone regions.
		- Generally lacking in disrupted regions.
		- Varying degrees in porous region.
Distribution of Calcite	- Medullary cavity-filling phase.	- Larger pores, gaps, and cracks.
	- Larger pores, gaps, and cracks.	- Calcite of varying thicknesses penetrating the bone wall.
Distribution of Clay Phases	A small number of minuscule pores are filled mostly with illite followed sparsely by vermiculite from the inner compact bone wall and cancellous bone.	- Medullary cavity-filling phase.
		- Porous region occupied by illite, kaolinite, vermiculite, and smectite.
		- A small number of minuscule pores are filled with illite from the inner compact bone wall.
Brief Interpretation	- Initial lithification likely occurred on or near the surface. Osteohistology preserved owing to the lack of extensive pressure and other interruptions.	- The combination of increase in size and crystallinity of apatite, decrease of open spaces between apatite crystals from pressure may have resulted in high level of nanostructural preservation.
	- Nanostructural preservation highly uneven, which suggested relatively more “open spaces” and slower diagenesis rate of apatite crystals.	- The overall density of the highly porous area was increased by the association of clay.
		- Calcite intrusion has damaged both the microstructure and nanostructure of bone in affected regions.
Evidence based on correlative microscopy	SEM micrographs show that although apatite density from the outer bone wall appears to be even in low magnifications, it is notably uneven in high magnifications, which correlates with the TEM data.	OM and SEM micrographs show the extent of relatively intact and disrupted bone tissues, and SAED patterns of apatite crystals from each corresponding region directly indicates the level of nanostructural preservation.
Issues	Our assumption on microstructural preservation may apply to the femur as the bone is composed of dense apatite, but we are uncertain if it also applies on bones with lesser density.	SAED data size is too small from porous region due to difficulty of obtaining FIB-milled samples with sufficient amount of apatite crystals because of its patchy distribution.
