# Supplementary material for: Disparities in correlating microstructural to nanostructural preservation of dinosaur femoral bones
Source: Sci Rep. 2017 Mar 30;7:45562. doi: 10.1038/srep45562 (PMC5372082; doi:10.1038/srep45562)
Supplement: Supplementary Information [file srep45562-s1.pdf]

Supplementary Information

Disparities in correlating microstructural to nanostructural perservation of dinosaur femoral bones

Jung-Kyun Kim, Yong-Eun Kwon, Sang-Gil Lee, Ji-Hyun Lee, Jin-Gyu Kim, Min Huh, Eunji Lee & Youn-Joong Kim

Supplementary Figures

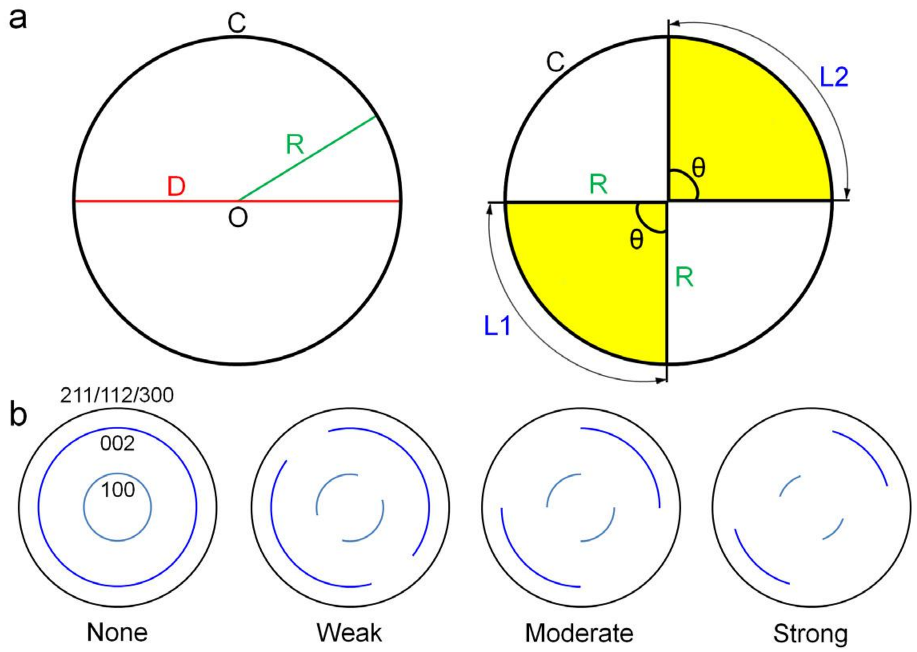

Figure S1. (a) Measurement values were acquired from the circumference lengths (C) of the {100} and {002} diffraction rings, and from their diffraction arc lengths (L) in each side, thus L1 and L2. The ratio of (L1+L2)/C were calculated and its values were used to evaluate the level of preferred orientation of apatite crystals from selected area electron diffraction (SAED) patterns. D = diameter, O = centre or origin, R = radius. (b) Representative pattern trends of the {100} and {002} diffraction rings based on the level of preferred orientation of apatite crystals. See Supplementary Table S2 for details on the measurement values.

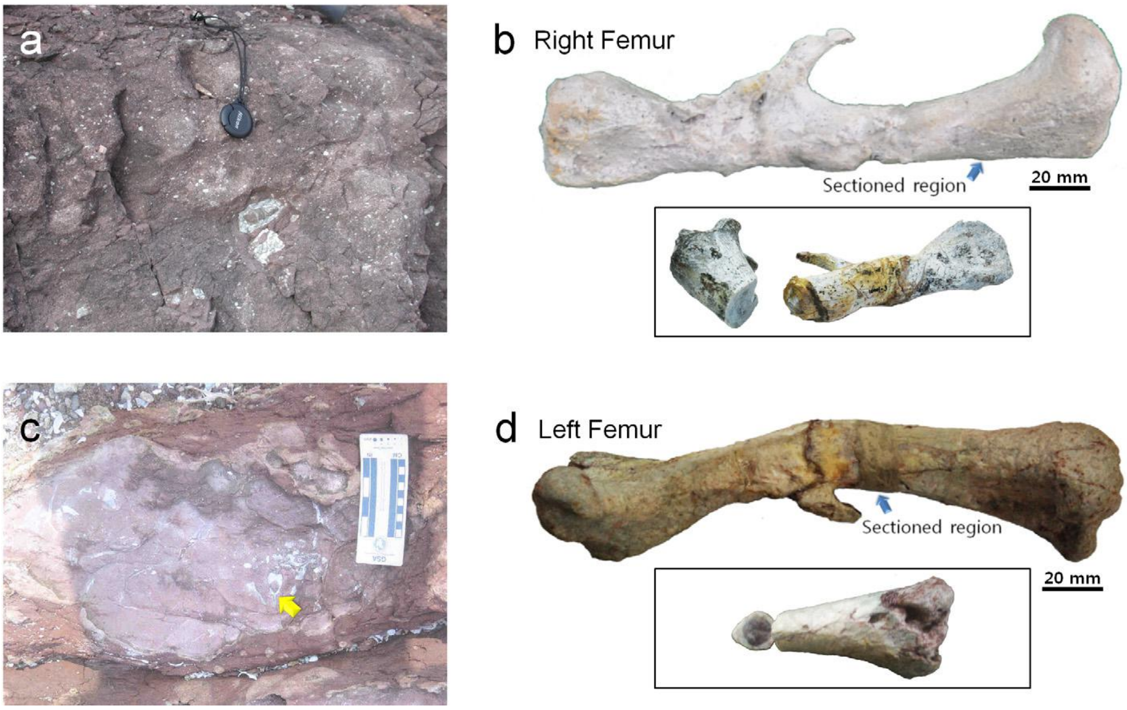

Figure S2. (a) Surface of the outcrop with rich calcite content where the right femur was discovered during excavation of egg fossils. The photo shows exposed indeterminate bone fragments. Camera lens cap diameter = 6 cm. (b) Medial view of the right femur. (c) Surface of the outcrop after initial excavation where the left femur and associated skeletal elements were discovered. The sacral vertebra can be identified (arrow). (d) Medial view of the left femur. Insets represent the current state of both femora after sectioning.

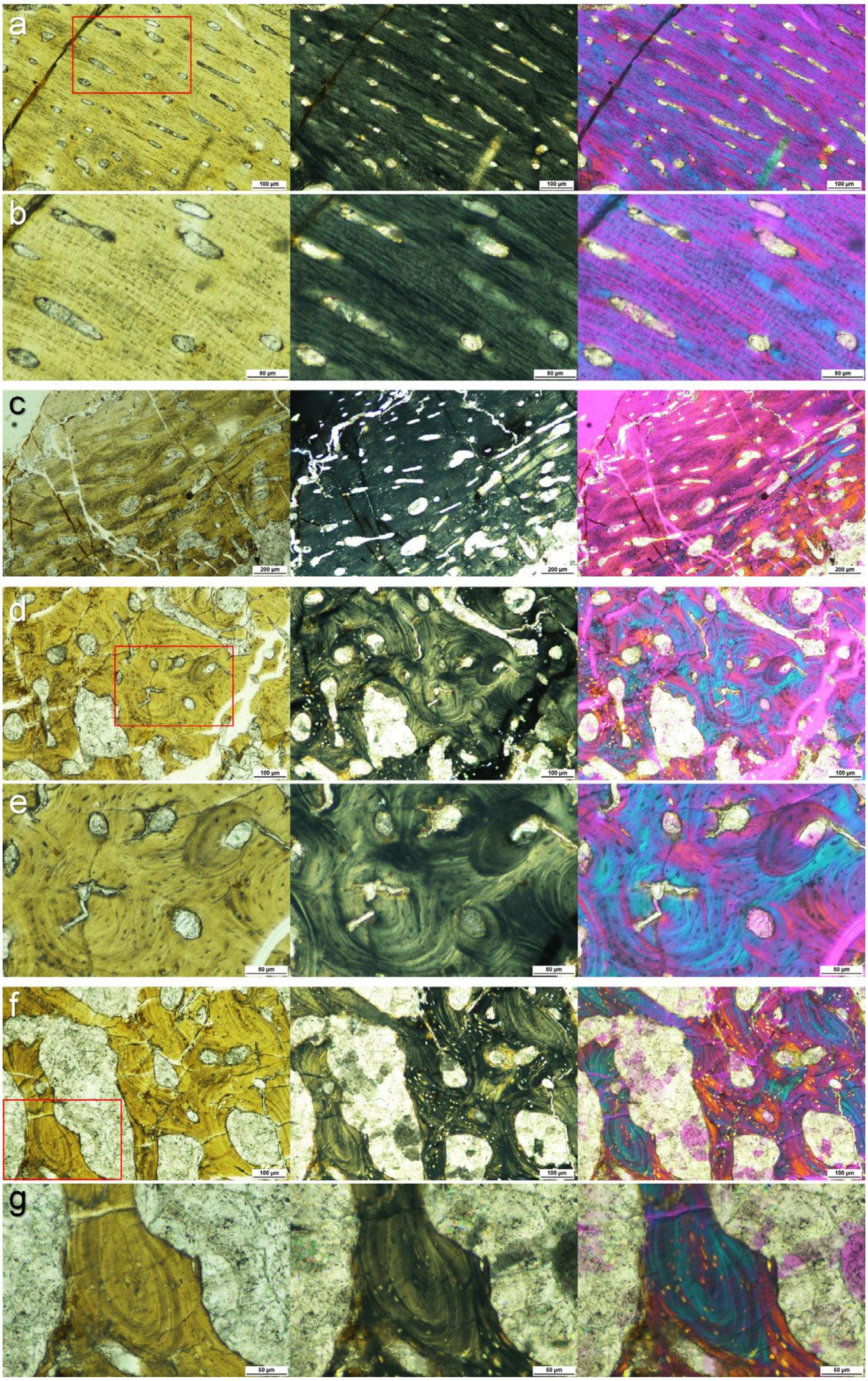
a
b
c
d
e
f
g

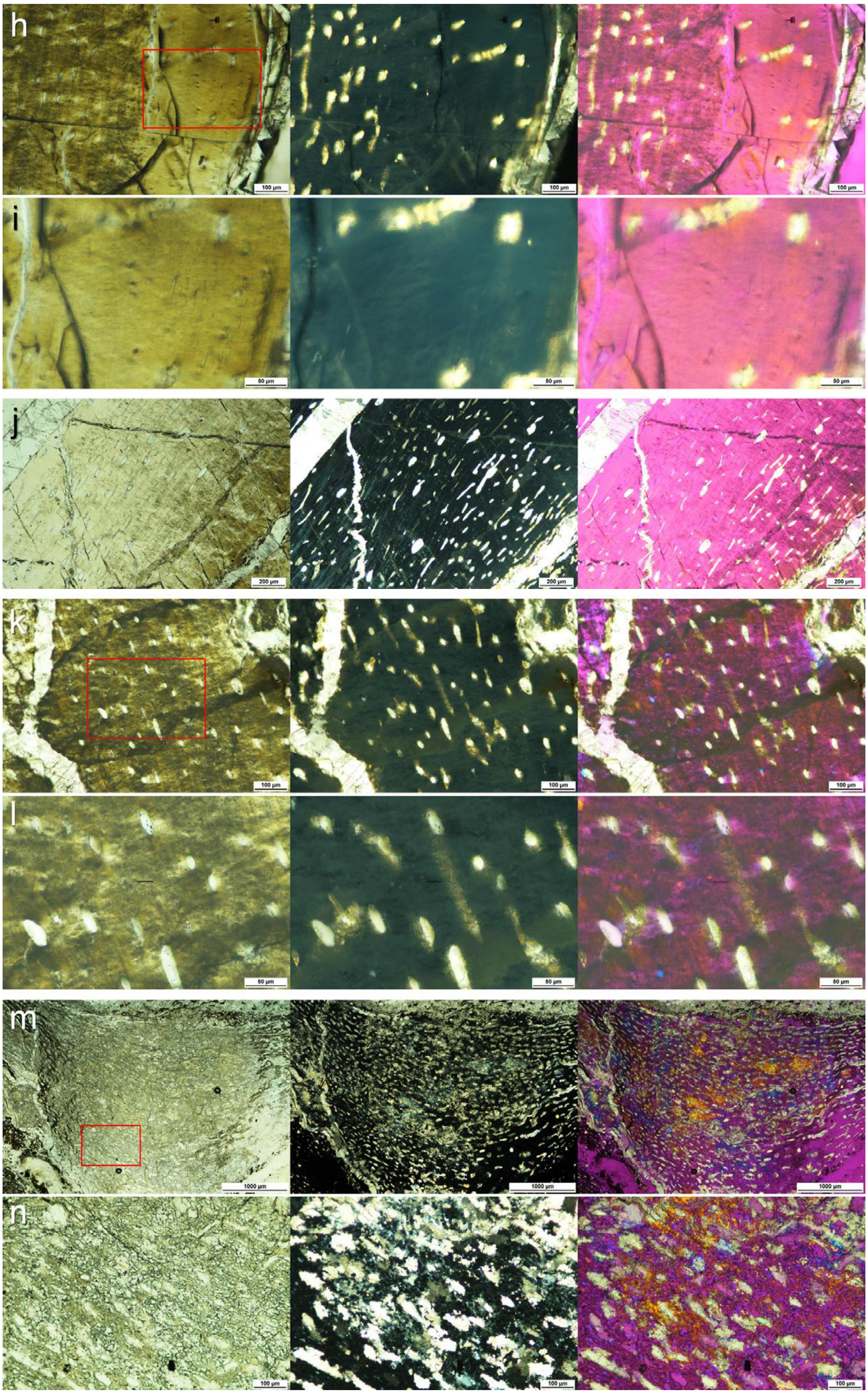

Figure S3. Optical micrographs highlighting the microstructures of both femora using normal transmitted light, cross-polarized light, and with the lambda wave plate (530 nm) inserted. (a~g) Right femur. (a) Outer bone wall. (b) Magnified image of the marked region in (a). (c) Outer to inner bone wall. (d) Middle bone wall with secondary osteons highlighted. Resorption spaces can be observed from these regions. (e) Magnified image of the marked region in (d). (f) Innermost bone wall with intact cancellous bone. (g) Magnified image of the marked region in (f). (h~n) Left femur. (h) Outermost bone wall. (i) Magnified image of the marked region in (h). (j,k) Inner bone wall with increased vascularity. (l) Magnified image of the marked region in (k). (m) Inner bone wall of the keeled region. (n) Magnified image of the marked region in (m).

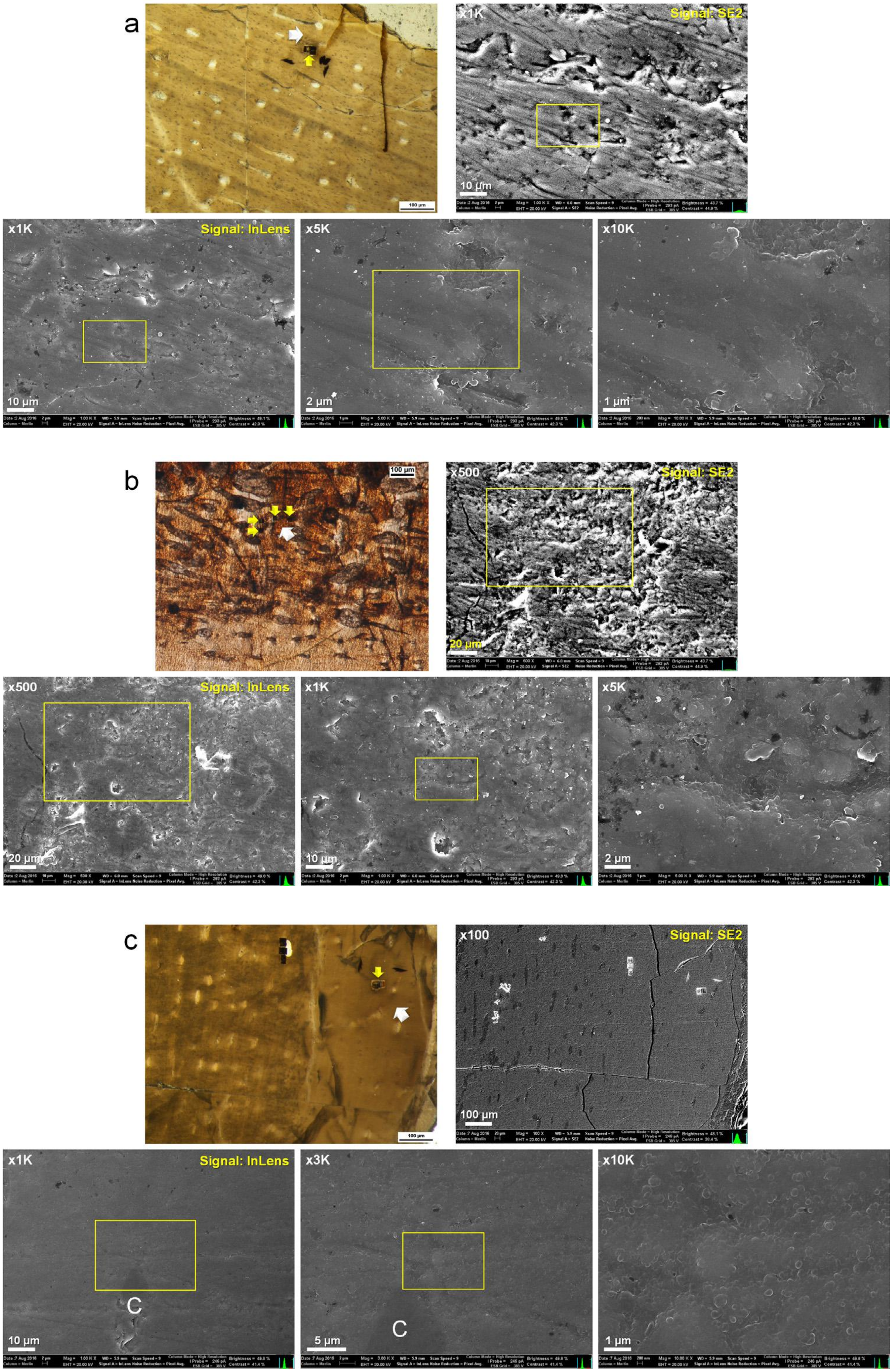
a
100 µm
x1K
Signal: SE2
10 µm
x1K
Signal: InLens
10 µm
x5K
2 µm
x10K
1 µm
b
100 µm
x500
Signal: SE2
20 µm
x500
Signal: InLens
20 µm
x1K
10 µm
x5K
2 µm
c
100 µm
x100
Signal: SE2
100 µm
x1K
Signal: InLens
C
10 µm
x3K
C
5 µm
x10K
1 µm

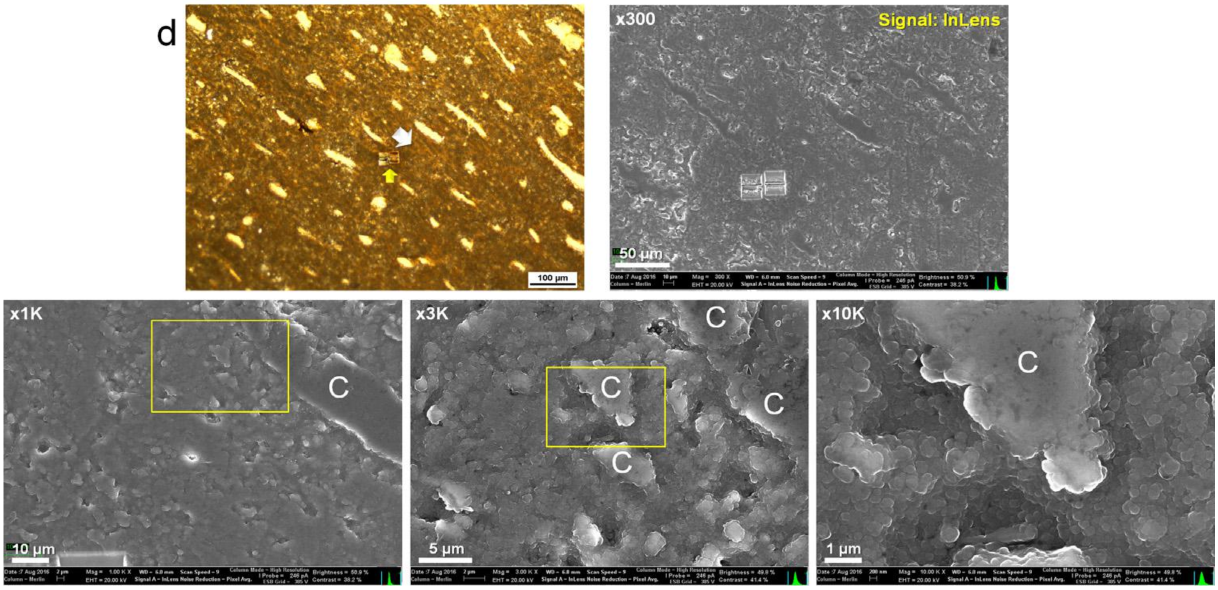

Figure S4. Scanning electron microscopy (SEM) imaging of both femora. In the optical micrographs, the white arrows indicate the region for SEM imaging, and the yellow arrows indicate areas where focused ion beam (FIB)-milled samples were obtained. (a,b) Right femur. (a) Outer bone wall. (b) Inner bone wall. Although the regions with dense apatite population appears to have a less rough surface in low magnifications, the overall variation of apatite density resulted in an uneven surface texture even from regions almost exclusively composed of apatite. (c,d) Left femur. (c) In the regions with microstructural features still relatively intact, the overall density of apatite are generally consistent, thus resulting in a smooth surface texture of the bone wall, even in high magnification SEM micrographs. (d) Bone matrix regions highly affected by calcite lose this feature, and shows that the surface morphology has become very uneven. C = calcite. Correspondence with Fig. 2: (a) with Fig. 2a, (b) with Fig. 2b, (c) with Fig. 2e, (d) with Fig. 2c.

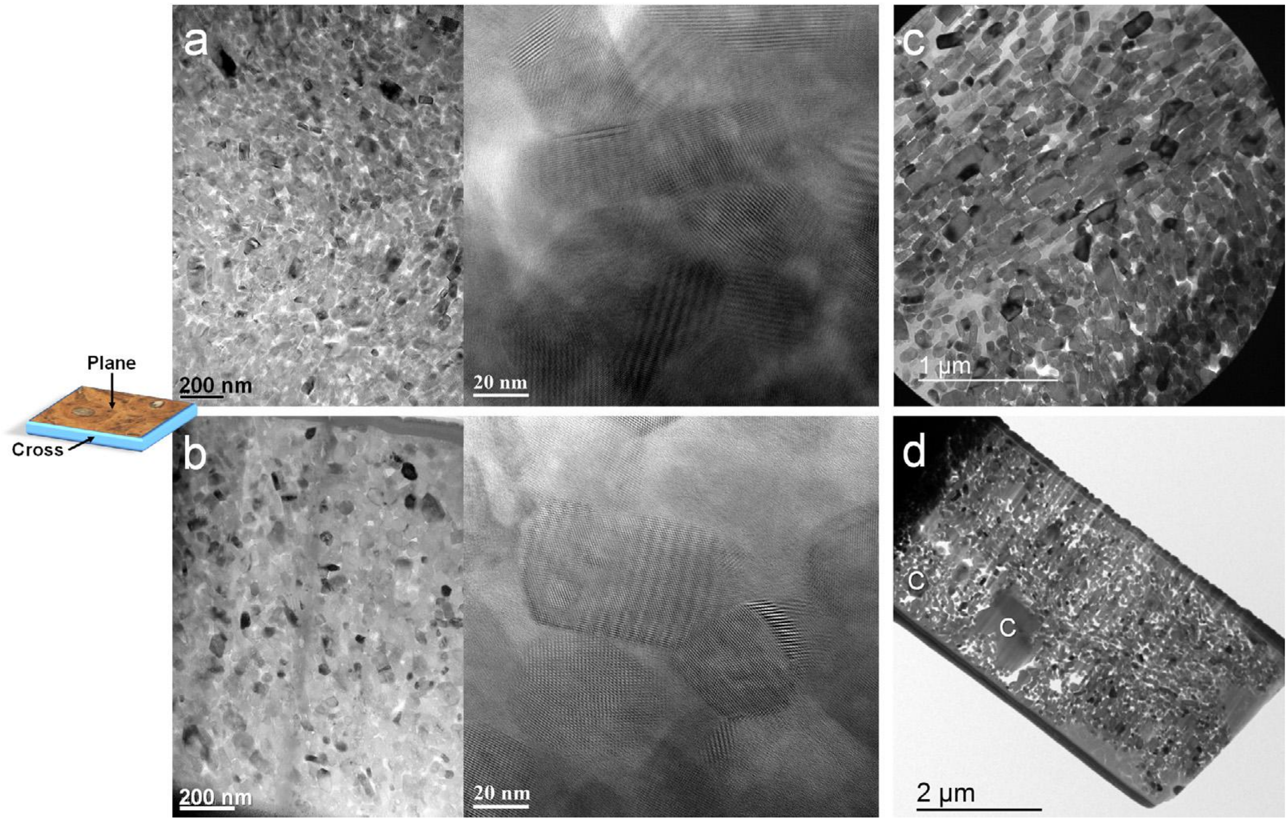

Figure S5. Representative transmission electron microscopy (TEM) micrographs of apatite crystals from FIB-milled samples. The subhedral to euhedral platelet-like morphology, and increased short-axis length can be observed. (a,b) Right femur samples from the outer bone wall region with associated high-resolution TEM (HRTEM) micrographs of the apatite crystals. (a) Cross-FIB-milled sample. (b) Plane-FIB-milled sample. (c,d) Left femur. (c) Cross-FIB-milled sample from the anterior inner porous region with the selected area (SA) aperture inserted. (d) Plane-FIB-milled sample from the inner bone wall of the posterior region. C = calcite.

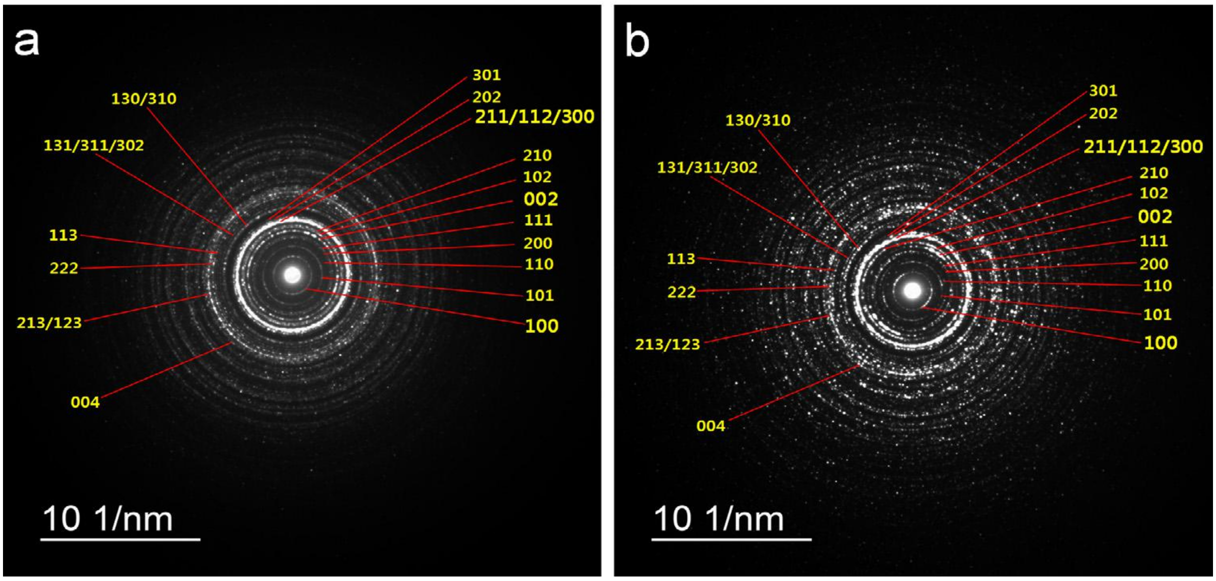

Figure S6. Representative SAED pattern indexing of both femora from cross-FIB-milled samples. (a) Right femur. (b) Left femur.

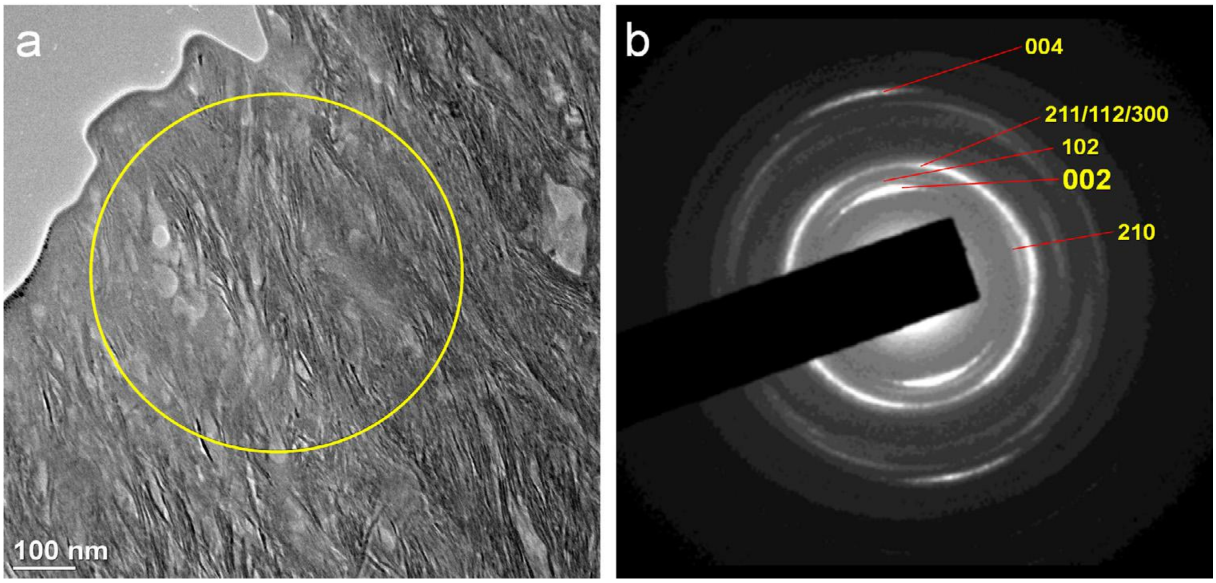

Figure S7. (a) TEM micrograph obtained from a cross-FIB-milled sample from an extant mouse femur (milled at the mid-diaphyseal region from an eight-week old female ICR [Imprinting Control Region] mouse) with the area designated for SAED pattern analysis indicated by the yellow circle. (b) The SAED pattern of the mouse femur shows that the certain sampled regions of the left femur likely retain its original nanostructure.

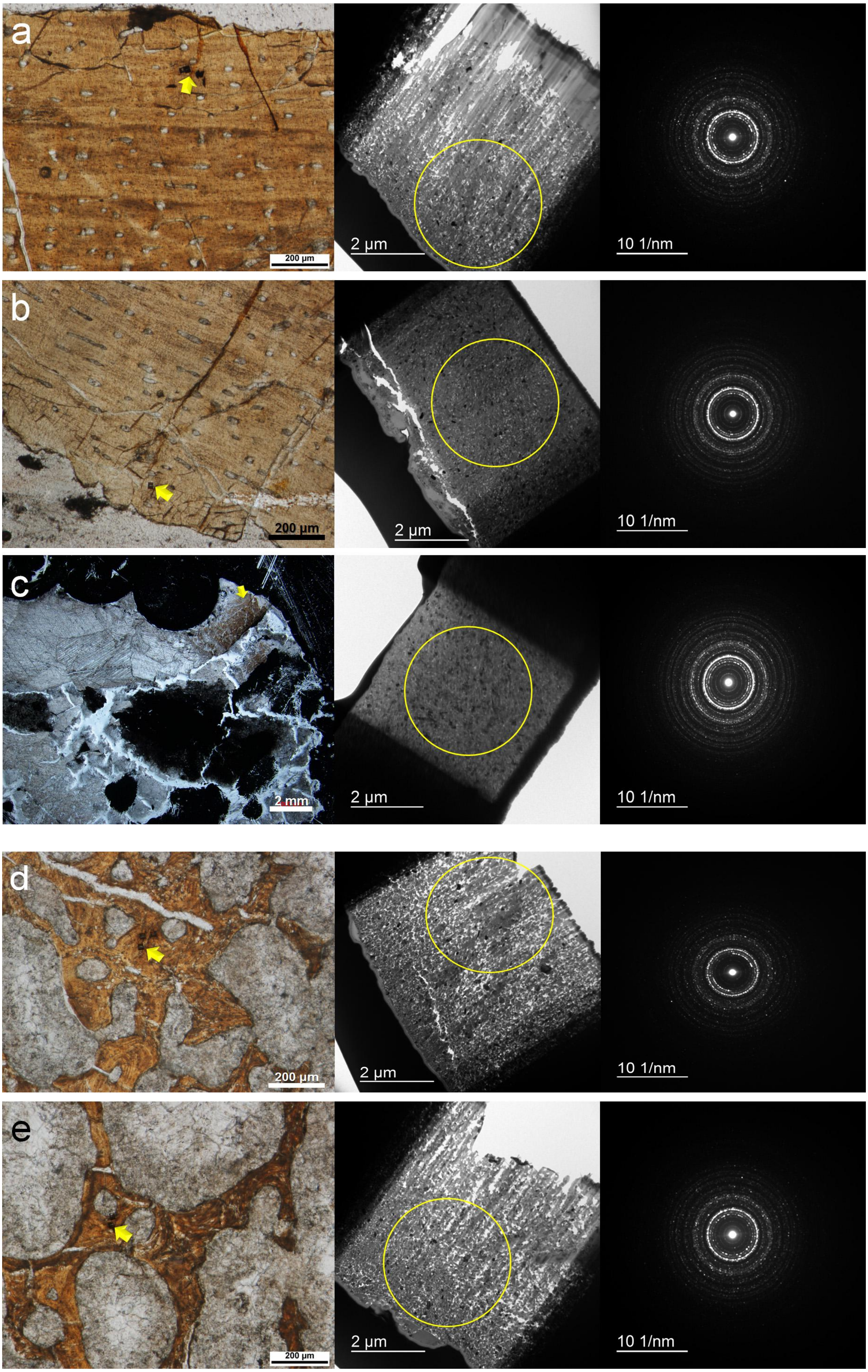
a
200 µm
2 µm
10 1/nm
b
200 µm
2 µm
10 1/nm
c
2 mm
2 µm
10 1/nm
d
200 µm
2 µm
10 1/nm
e
200 µm
2 µm
10 1/nm

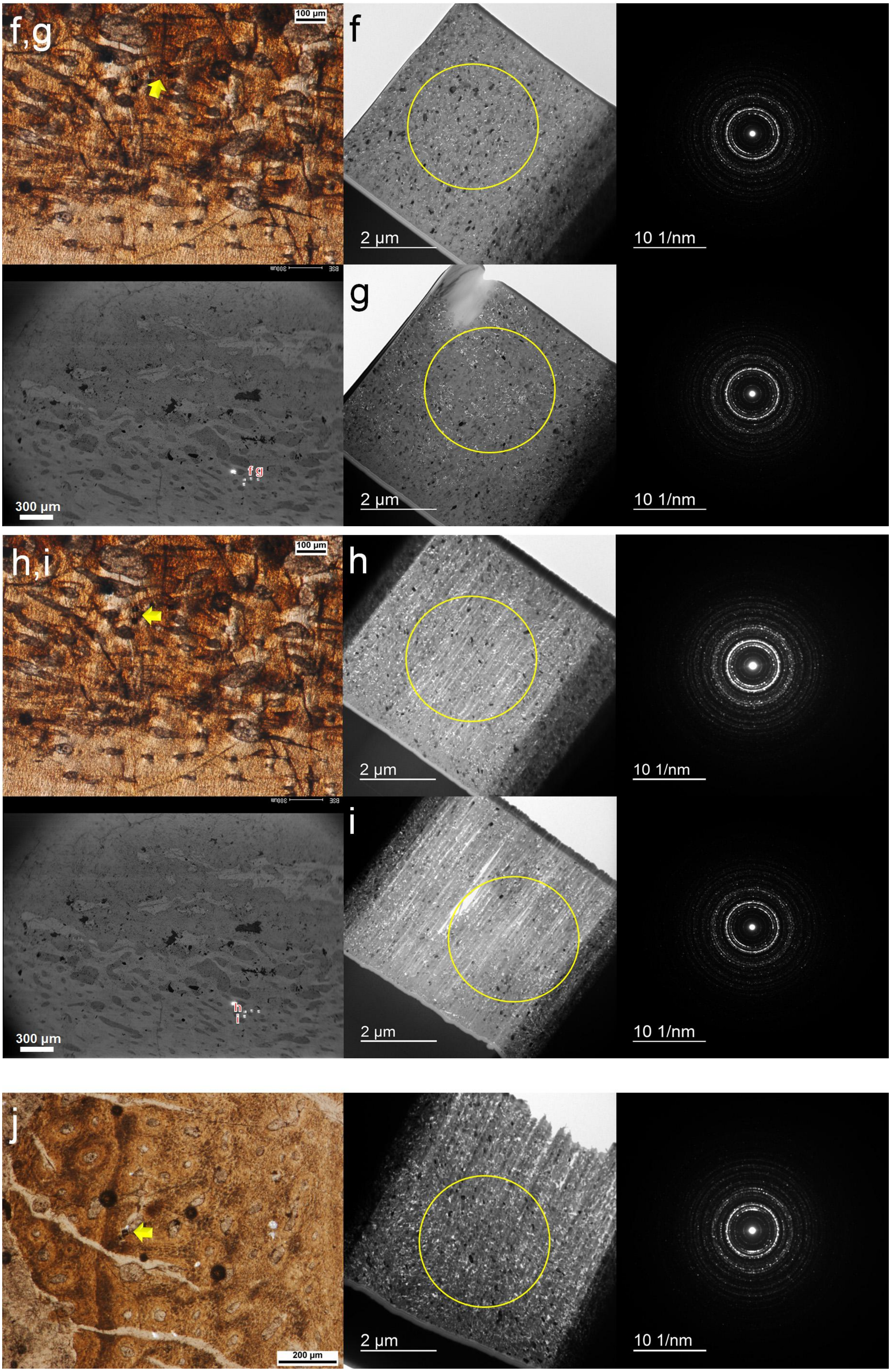

f,g
100 µm
f
2 µm
10 1/nm
g
2 µm
10 1/nm
300 µm
f g
h,i
100 µm
h
2 µm
10 1/nm
i
2 µm
10 1/nm
300 µm
h
i
j
200 µm
2 µm
10 1/nm

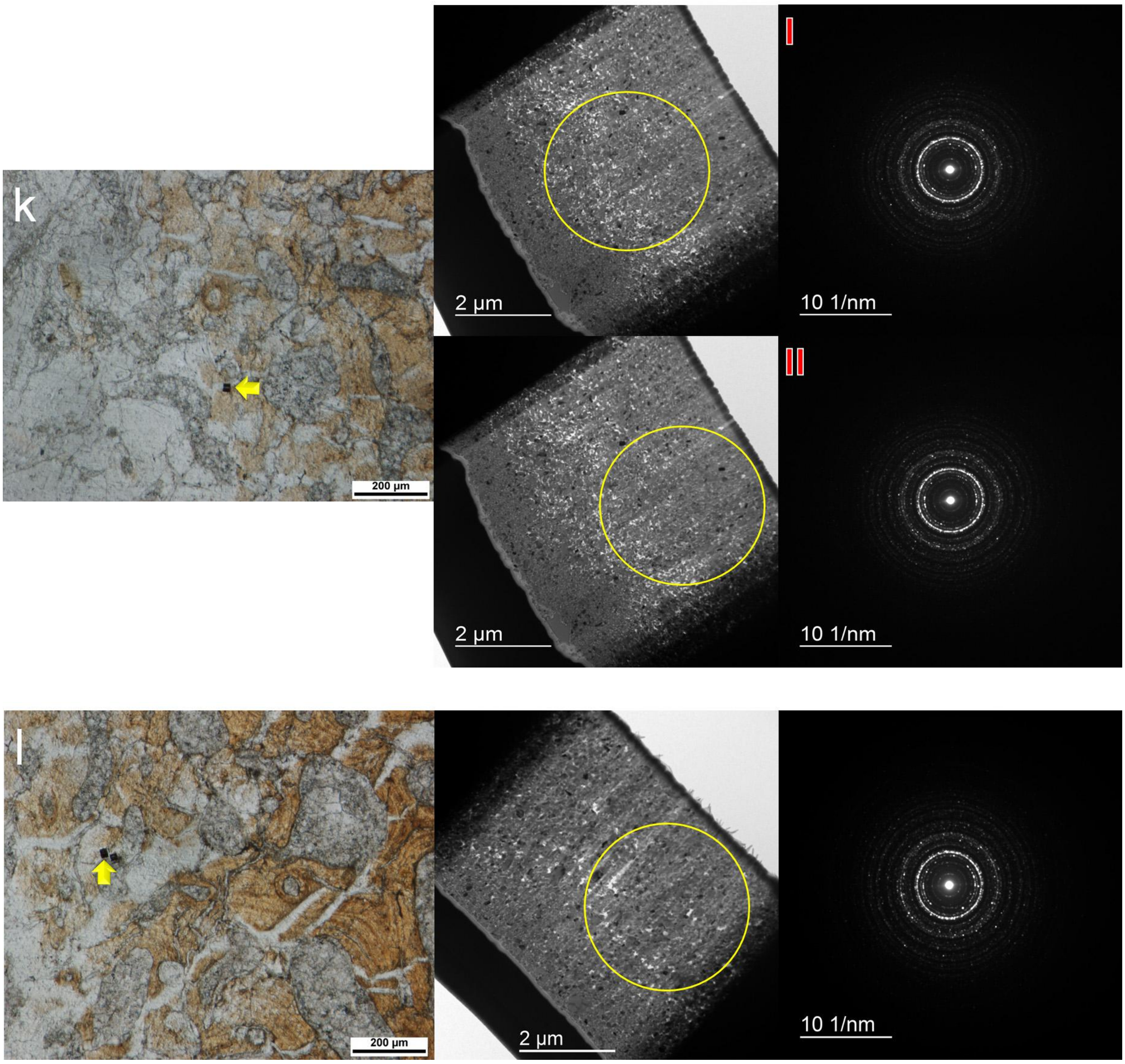

Figure S8. Exact location of cross-FIB-milled samples in the right femur from Figure 3, and corresponding TEM micrographs with the designated areas in yellow circles for SAED pattern analysis. (a-c) Outermost bone wall. (d,e) Innermost bone wall with cancellous bone. (f-l) Inner bone wall.

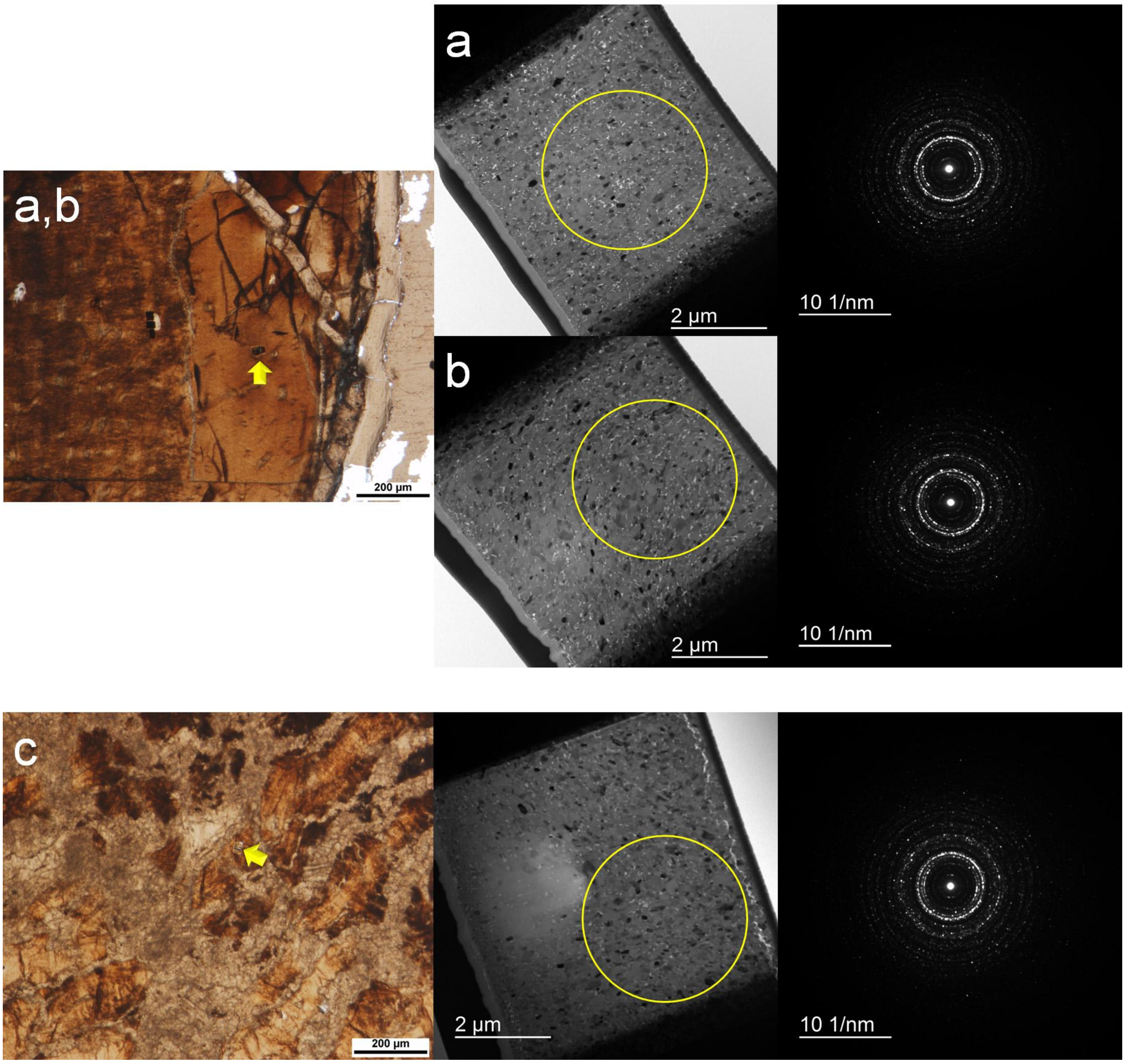
a,b
200 µm
a
2 µm
10 1/nm
b
2 µm
10 1/nm
c
200 µm
2 µm
10 1/nm

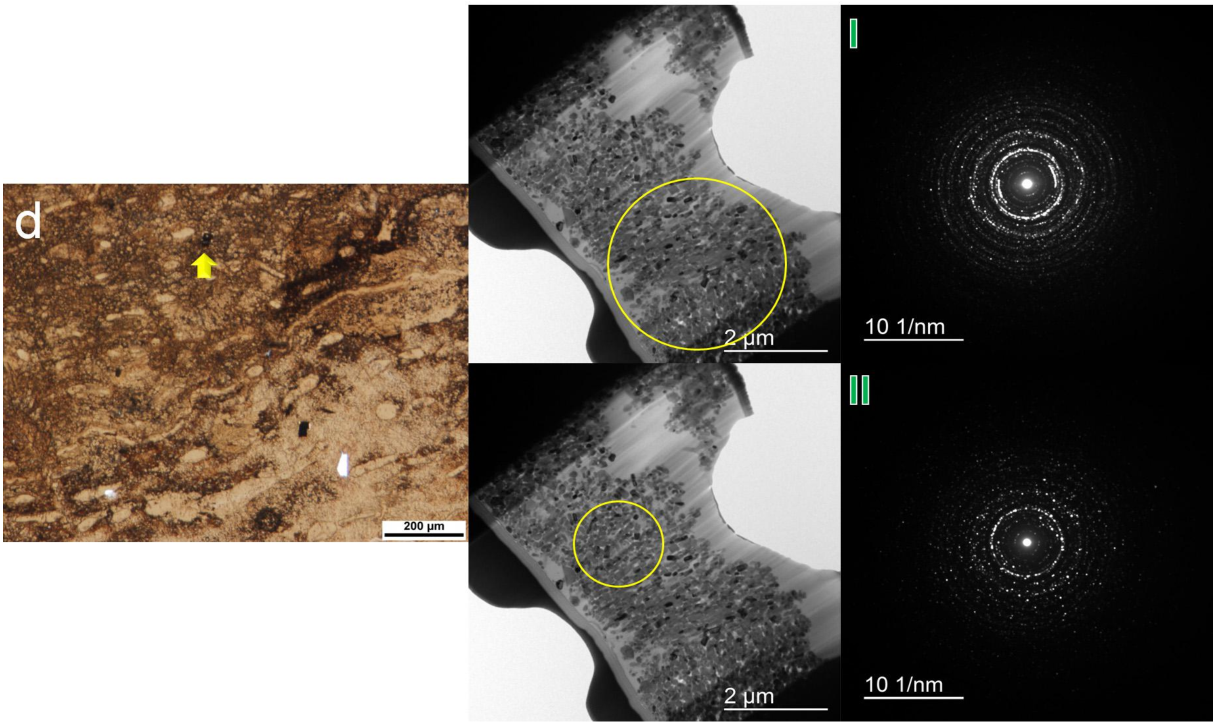
d
200 µm
I
II
2 µm
10 1/nm

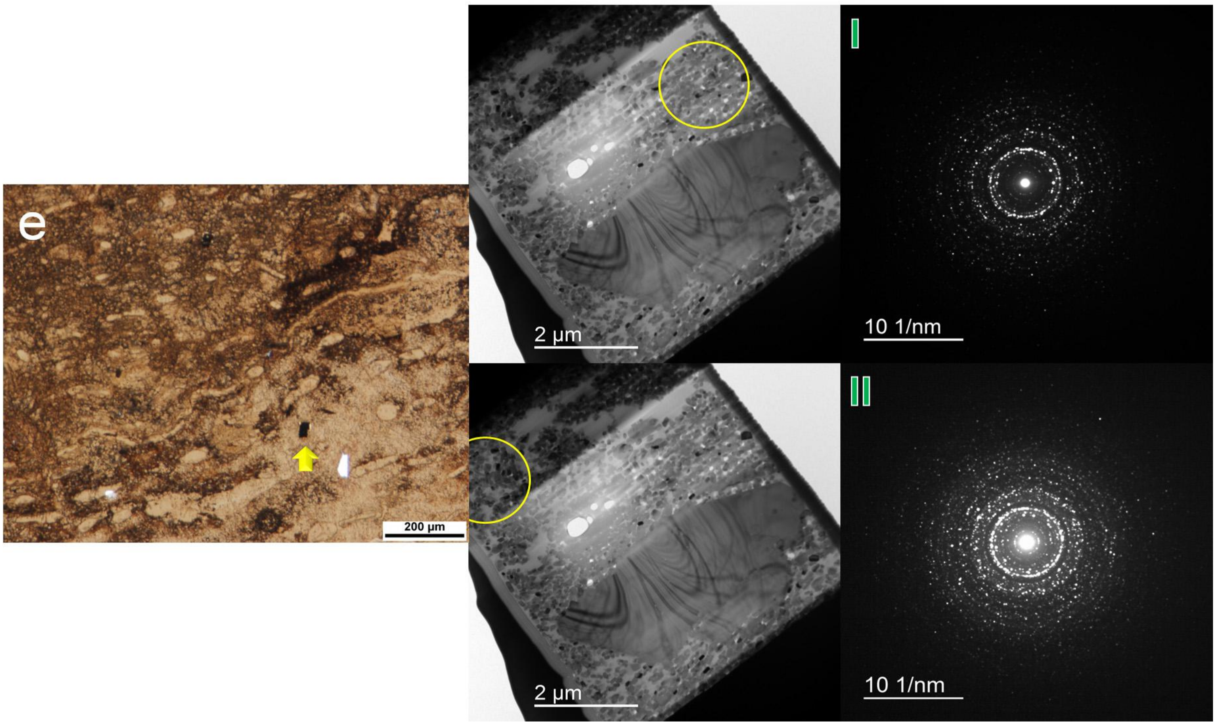
e
200 µm
I
II
2 µm
10 1/nm

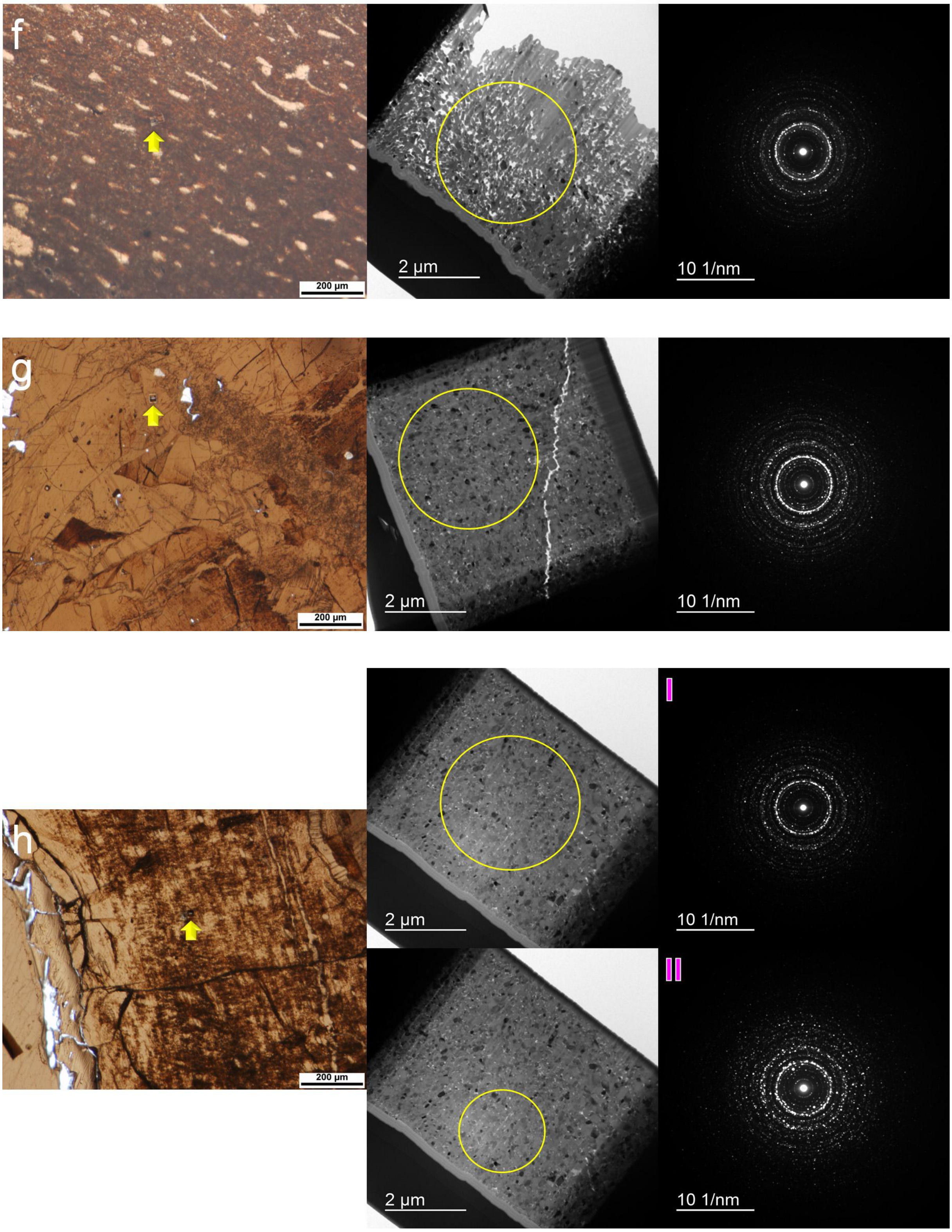
f
200 µm
2 µm
10 1/nm
g
200 µm
2 µm
10 1/nm
I
h
200 µm
2 µm
10 1/nm
II
2 µm
10 1/nm

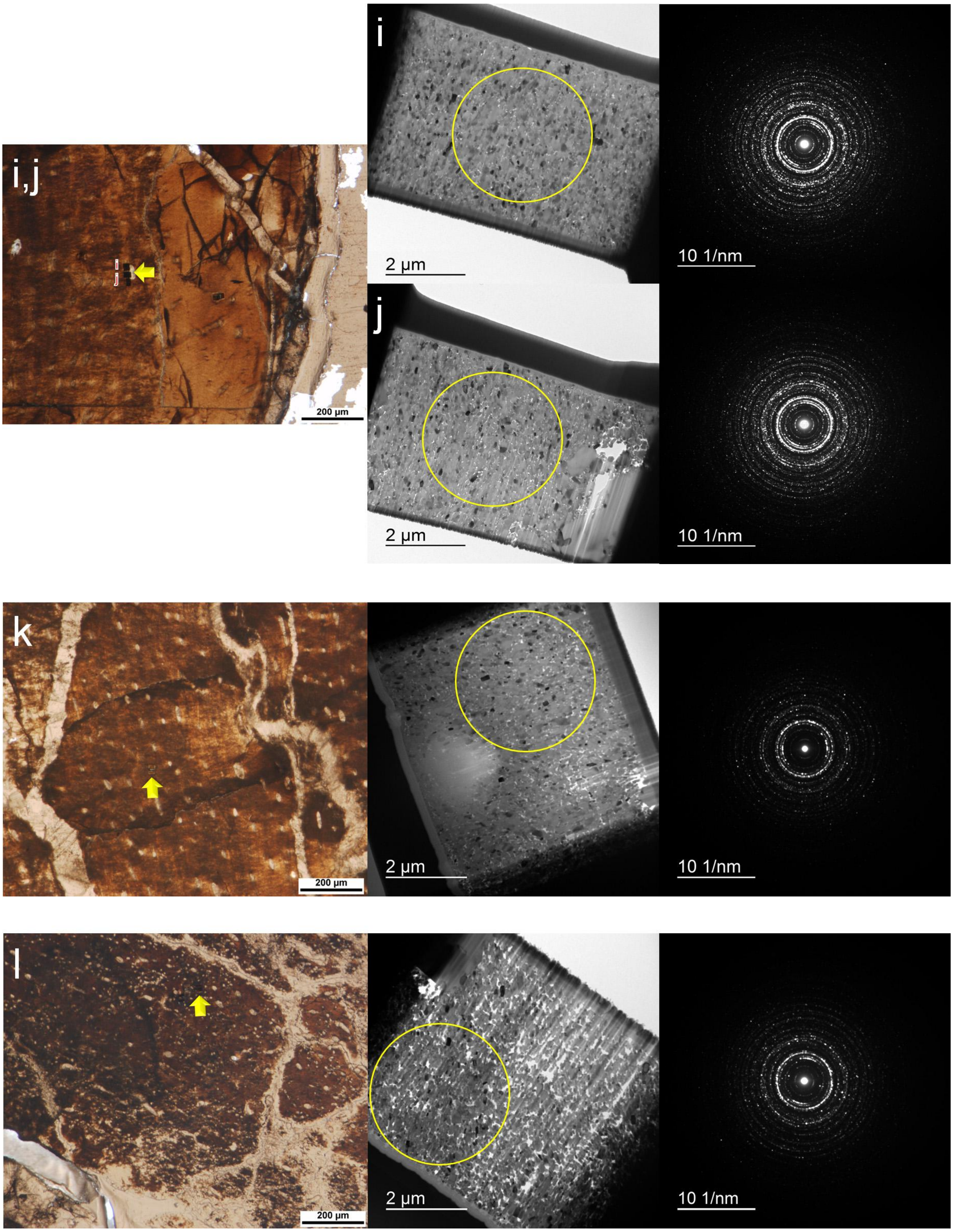

Figure S9. Exact location of cross-FIB-milled samples in the left femur from Figure 4, and corresponding TEM micrographs with the designated areas in yellow circles for SAED pattern analysis. (a,b) Outermost bone wall. (c) Innermost bone wall. (d,e) Anterior inner porous region. (f,g) Inner bone wall disrupted by calcite. (h) Disrupted inner bone wall. (i-l) Inner bone wall.

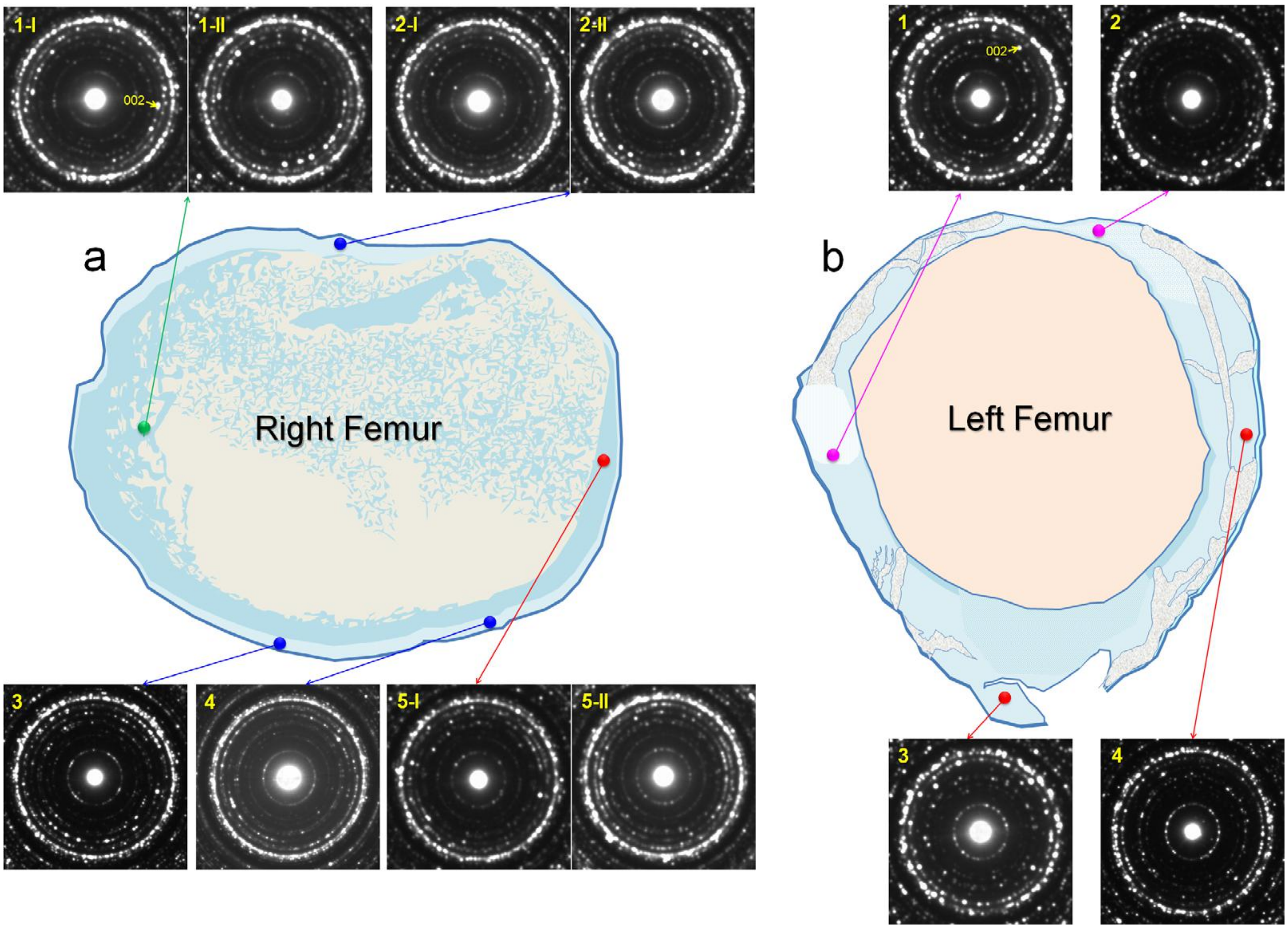

Figure S10. SAED patterns of the plane-FIB-milled samples from both femora. (a) Right femur. (b) Left femur. The arrangement of apatite crystals appeared to be generally random with the exception from a sample obtained directly from an osteon within the right femur (a1-I and a1-II; green dot, arrow), and inner bone wall samples (b1 and b4) from the left femur which all had weak degrees of preferred orientation. The intensity of the {002} plane from all the left femur samples were very weak.

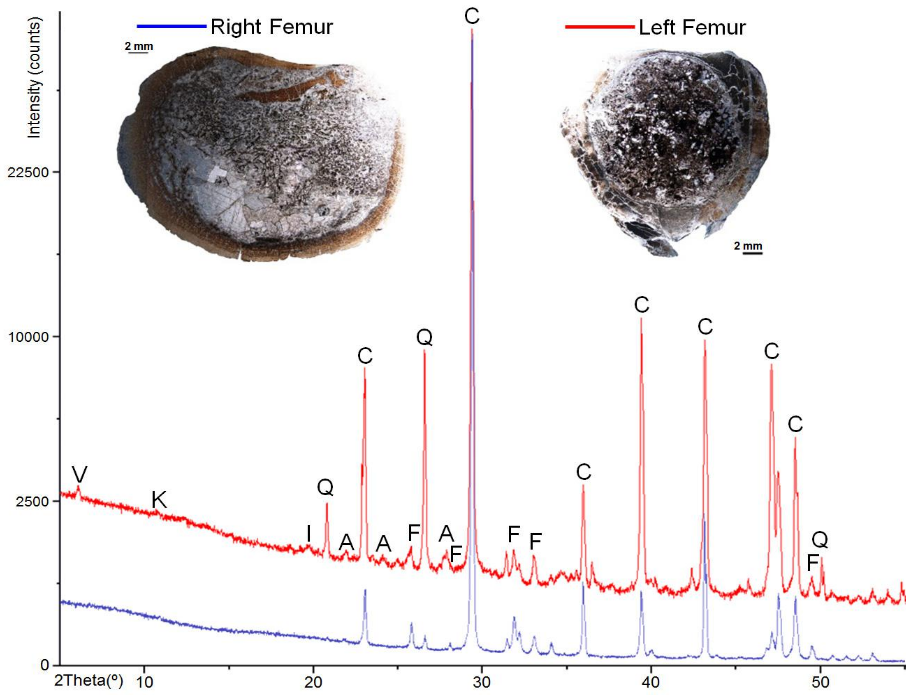

Figure S11. X-ray Diffraction (XRD) analysis for identifying phases from optical thin sections of both femora. Main phases directly from the bone matrices are fluorapatite, calcite, and quartz. From the medullary cavity of both femora, the right femur is mainly filled with calcite, and the left femur is filled with mudstone along with small detrital quartz and albite clasts. The clay peaks are primarily detected from this region. A = albite, C = calcite, F = fluorapatite, I = illite, K = kaolinite, Q = quartz, V = vermiculite.

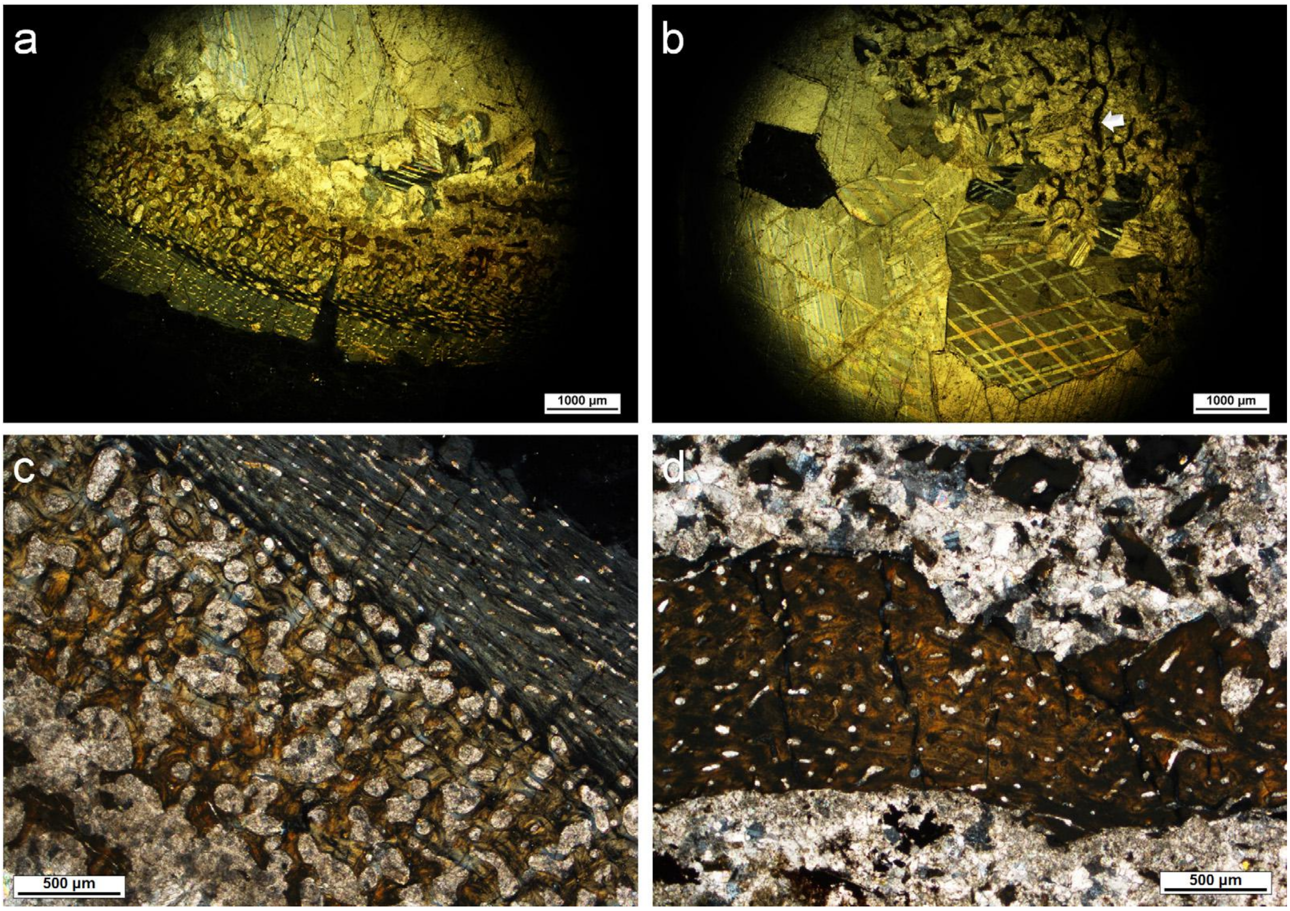

Figure S12. Cross-polarized optical micrographs of calcite within the right femur. The yellowish tint in (a,b) is due to the carbon coating. (a) Overall appearance of calcite from the bone wall and medullary cavity. (b) Calcite within the medullary cavity. Calcite appears as clusters of microcrystals in regions with cancellous bone fragments (arrow), and as a group of larger phenocrysts in regions lacking bone fragments. (C) Clusters of microcrystalline calcite in vascular channels and resorption spaces. (d) Clusters of microcrystalline calcite, and the relatively well-preserved osteohistological features from the broken off inner bone wall layer can be observed.

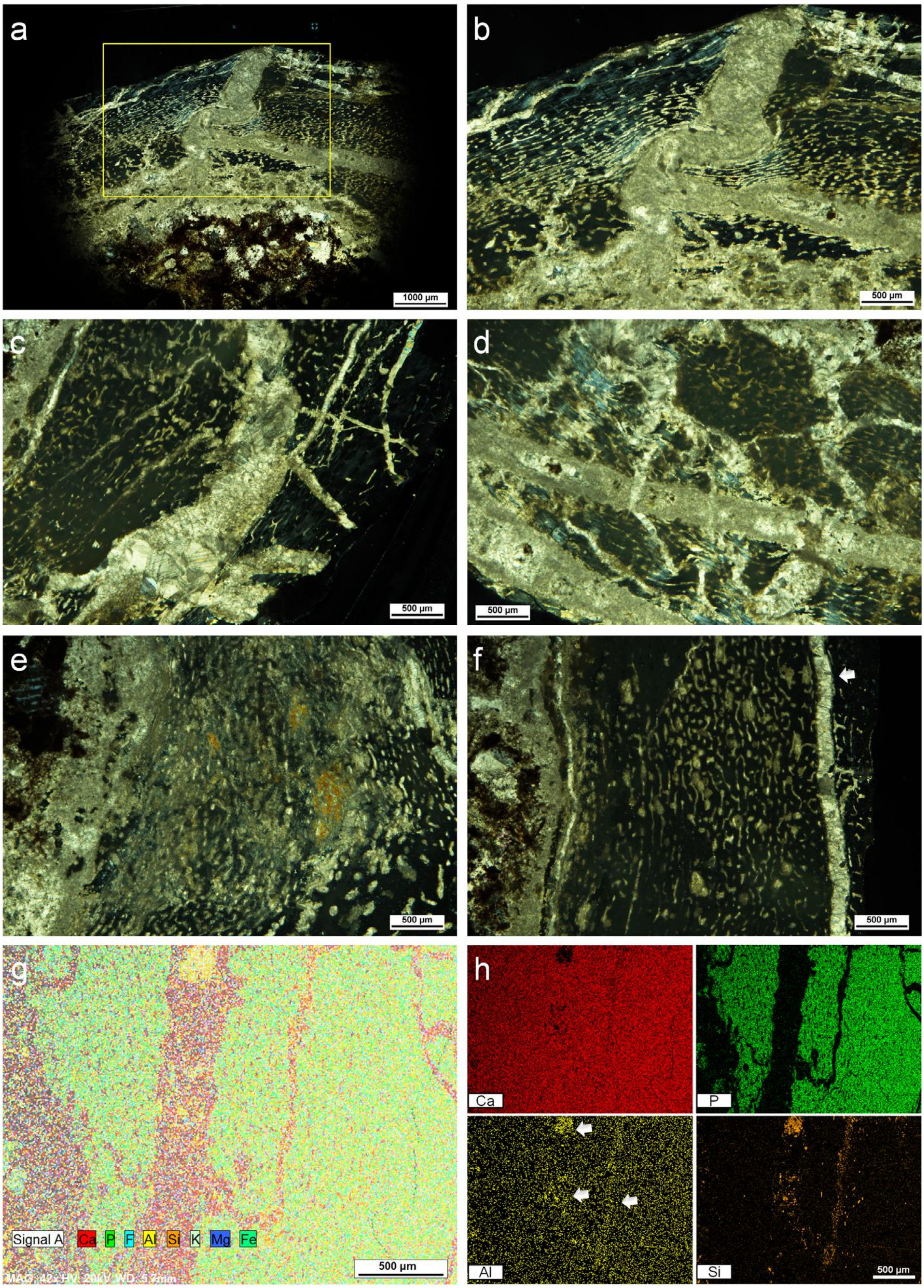

Figure S13. Cross-polarized optical micrographs of calcite within the left femur displaying the uneven size distribution of calcite crystals. (a) Calcite intrusion affecting the bone wall when the bone was in a relatively fresh state. (b) Magnified image of the squared region in (a). (c,d) Calcite intrusion in different scales within the outer bone wall. (e) Calcite distribution in the anterior porous region. (f) Calcite intrusion with distinct clusters of microcrystals (arrow). (g,h) SEM-energy dispersive spectroscopy (EDS) mapping of intrusive calcite consisting individual calcite crystals at submicron scale. The presence of clay minerals detected in (g) is based on the overlapping distribution of Al and Si in (h). Note that the Al signals in (h) are mostly noise signals except for the areas overlapping with Si (arrows).

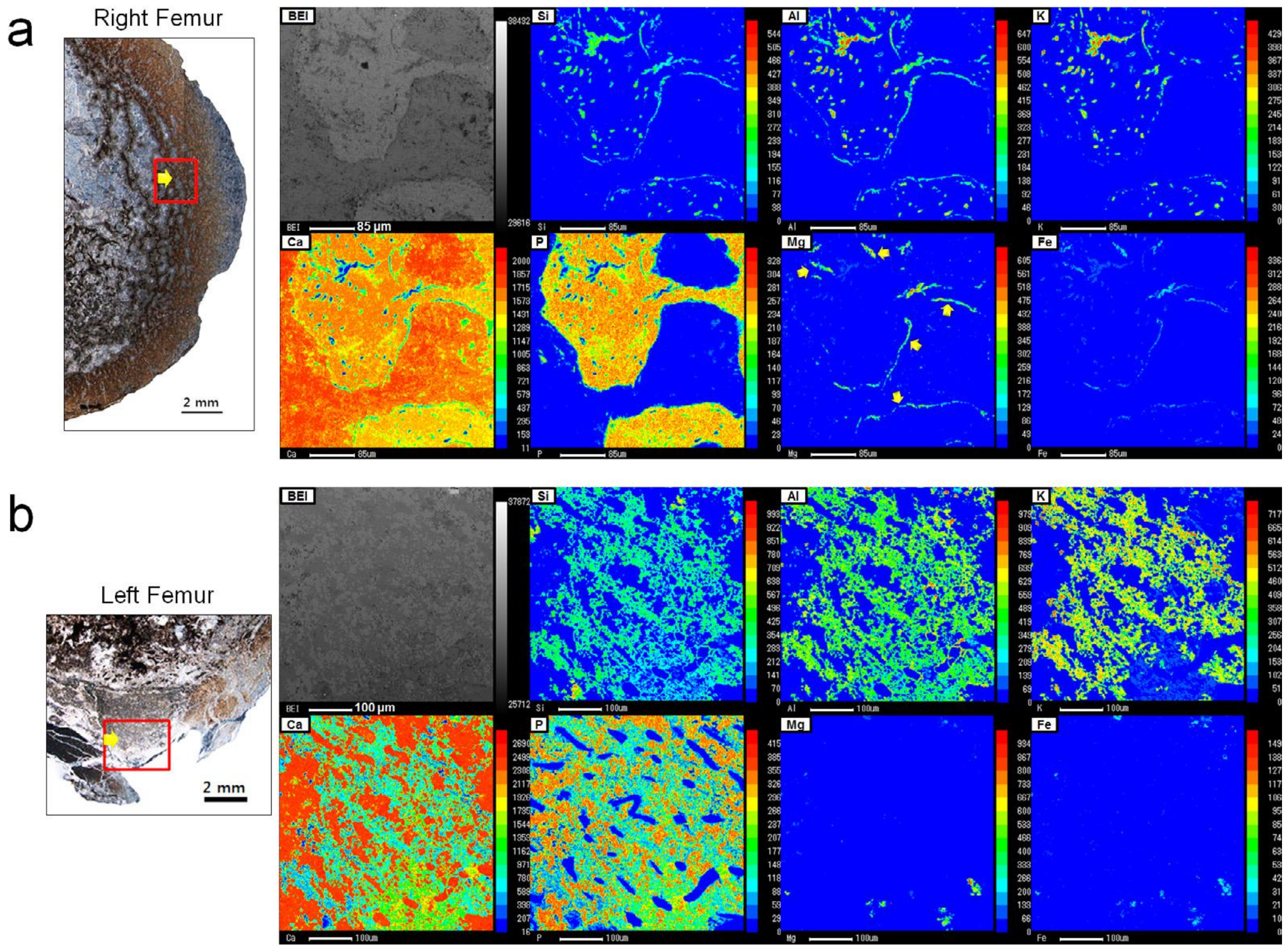

Figure S14. Electron probe microanalyzer with wavelength dispersive spectroscopy (EPMA-WDS) mapping of both femora from regions rich in illite. (a) The clay content of the right femur is sparse, and the innermost bone wall region from the lateral portion of the femur has the highest clay content which was mainly identified as illite. Vermiculite usually occurred around the outer rims of the bone matrix (arrows in Mg map). (b) The inner anterior porous region of the left femur is exceptionally rich in clay content, and the lower left portion has the highest illite concentration. BEI = backscattered electron image.

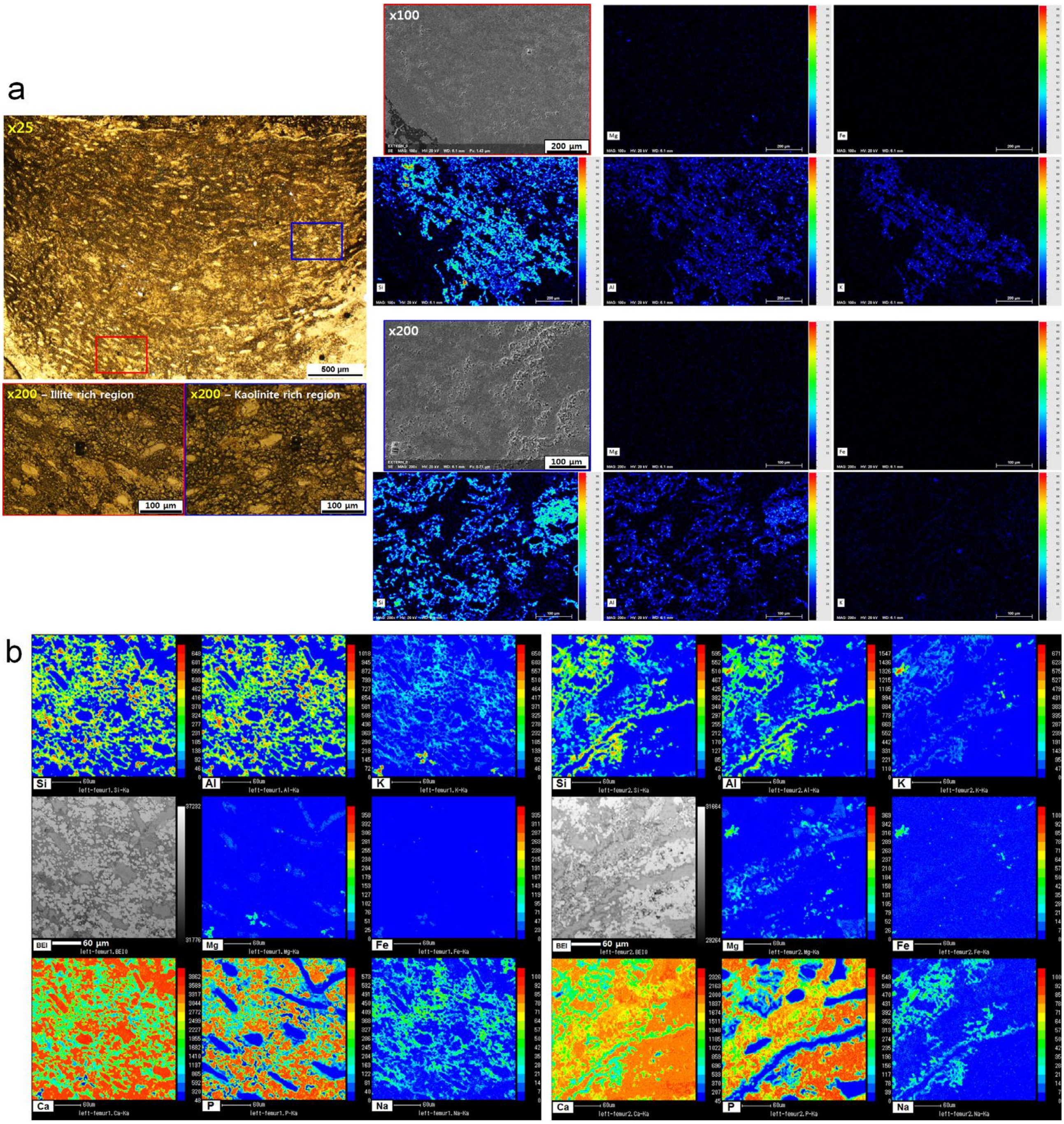

Figure S15. Clay phase distribution mapping on the anterior porous region of the left femur. (a) SEM-EDS mapping on regions rich in illite and kaolinite, respectively. (b) EPMA-WDS mapping for discerning less prominent clay phases and their distribution.

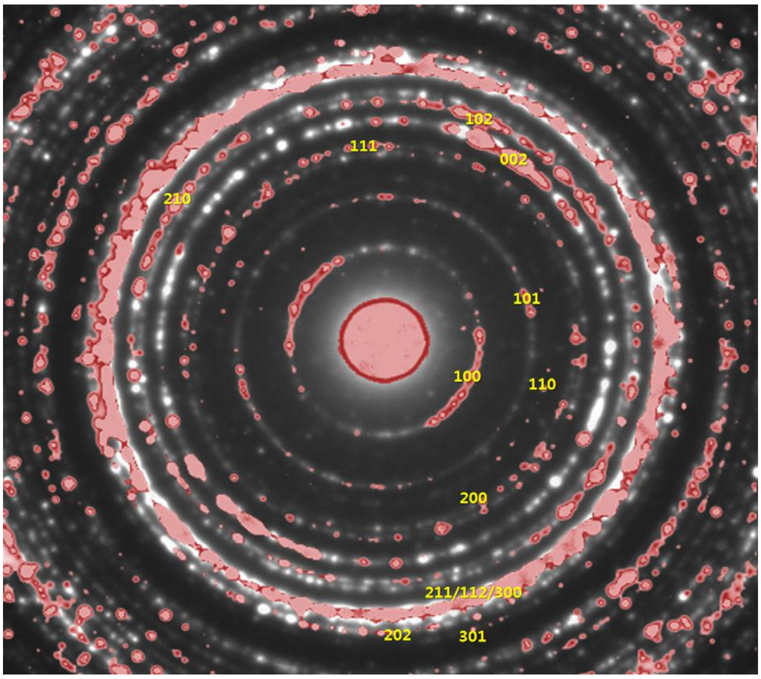

Figure S16. Overlapped inner ring SAED patterns from both femora (right - white, left - red). Indexing specific diffraction planes can be achieved more efficiently, and the consistency of the SAED pattern data obtained from different samples is shown.

Supplementary Tables

Supplementary Table S1: *Koreanosaurus boseongensis* specimens. All specimens were excavated and collected by the Korea Dinosaur Research Center team at Chonnam National University during 2003, from the Late Cretaceous dinosaur egg fossil sites (5 sites in total) located in the coastal areas of Bibong village, Boseong County, South Korea. The specimen numbers were designated as KDRC-BB# (Korea Dinosaur Research Center – Boseong Bibong #), and the order was based on time of discovery.

**Specimen number**	**KDRC-BB1**	**KDRC-BB2**	**KDRC-BB3**
**Specimen designation**	Referred	Holotype	Paratype
**Elements**	Right femur, proximal part of the left tibia, a caudal vertebra	Five cervical vertebrae, seven dorsal vertebrae, seventeen dorsal ribs and additional rib fragments, nearly complete right and left scapulae, coracoids, sternals, partial right humerus, proximal part of the left humerus, and proximal parts of the left ulna and radius	Left Femur, left tibia, left fibula, partial left metatarsals, partial left ilium, proximal part of the left ischium, and partial sacral vertebrae
**In articulation**	No	Yes	Yes
**Discovery Site**	Site 3	Site 5	Site 5
**Discovery region**	Lower mid region of the mudstone outcrop	Mudstone boulder detached from the mudstone outcrop	Base of the mudstone outcrop
**Initial preserved state**	Skeletal elements encased in calcite nodules, nodules embedded in mudstone	Skeletal elements directly embedded in mudstone	Skeletal elements directly embedded in mudstone

Supplementary Table S2: Arc length (L) / circumference length (C) ratio measurements from the {100} and {002} diffraction rings. We used the following ratio values for assessing the level of preferred orientation: 1.00 = No preferred orientation; 0.56 to 0.79 = Weak; 0.43 to 0.55 = Moderate; 0.42 and lower = Strong.

**Femur**	**{100}C**	**{100}L1**	**{100}L2**	**(L1+L2)/C**	**{002}C**	**{002}L1**	**{002}L2**	**(L1+L2)/C**	**Preferred Orientation**	**Notes**
**Right Femur**										
**Fig. 3a**	16.64	16.64		1.00	**38.97**	**38.97**		**1.00**	None	
**Fig. 3b**	16.64	6.01	5.96	0.72	**38.97**	**11.37**	**11.32**	**0.58**	Weak	Typical weak pattern
**Fig. 3c**	16.20	16.20		1.00	**37.99**	**37.99**		**1.00**	None	Pseudolamellar bone from upper thin section
**Fig. 3d**	16.96	3.49	3.53	0.41	**39.56**	**8.35**	**8.24**	**0.42**	Strong	{100} arc intensity weak
**Fig. 3e**	16.96	16.96		1.00	**39.56**	**39.56**		**1.00**	None	
**Fig. 3f**	16.96	5.70	5.70	0.67	**39.56**	**11.90**	**11.85**	**0.60**	Weak	{002} arc intensity strong, {100} very weak
**Fig. 3g**	16.96	16.96		1.00	**39.56**	**39.56**		**1.00**	None	
**Fig. 3h**	16.96	6.12	6.12	0.72	**39.56**	**9.89**	**9.89**	**0.50**	Moderate	{100} and {002} arc intensities strong
**Fig. 3i**	16.96	5.56	5.53	0.65	**39.56**	**10.22**	**10.22**	**0.52**	Moderate	{100} arc intensity weak
**Fig. 3j**	16.64	4.16	4.16	0.50	**38.97**	**6.98**	**6.98**	**0.36**	Strong	{100} and {002} arc intensities relatively strong
**Fig. 3kI**	16.64	16.64		1.00	**38.97**	**38.97**		**1.00**	None	
**Fig. 3kII**	16.64	16.64		1.00	**38.97**	**13.53**	**13.58**	**0.70**	Weak	{002} arc intensity uneven
**Fig. 3l**	16.64	5.59	5.57	0.67	**38.97**	**11.85**	**11.80**	**0.61**	Weak	{002} arc intensity uneven
**Left Femur**										
**Fig. 4a**	16.64	3.07	3.10	0.37	**38.97**	**7.31**	**7.31**	**0.38**	Strong	{100} arc intensity uneven
**Fig. 4b**	16.64	3.00	3.03	0.36	**38.97**	**8.12**	**8.12**	**0.42**	Strong	{100} arc intensity weak
**Fig. 4c**	16.64	4.16	4.16	0.50	**38.97**	**9.53**	**9.58**	**0.49**	Moderate	{100} arc intensity uneven
**Fig. 4dI**	16.64	3.21	3.24	0.39	**38.97**	**5.63**	**5.68**	**0.29**	Strong	From sufficient amount of apatite
**Fig. 4dII**	16.64	16.64		1.00	**38.97**	**38.97**		**1.00**	None	Apatite amount insufficient
**Fig. 4eI**	16.64	16.64		1.00	**38.97**	**38.97**		**1.00**	None	Apatite amount insufficient
**Fig. 4eII**	16.64	16.64		1.00	**38.97**	**38.97**		**1.00**	None	Apatite amount insufficient
**Fig. 4f**	16.64	4.95	4.92	0.59	**38.97**	**10.82**	**10.82**	**0.56**	Weak	Disrupted region, {002} arc intensity very weak
**Fig. 4g**	16.64	16.64		1.00	**38.97**	**38.97**		**1.00**	None	Disrupted region
**Fig. 4hI**	16.64	16.64		1.00	**38.97**	**38.97**		**1.00**	None	Disrupted region
**Fig. 4hII**	16.64	16.64		1.00	**38.97**	**38.97**		**1.00**	None	Disrupted region
**Fig. 4i**	16.77	3.38	3.40	0.40	**39.31**	**6.06**	**6.12**	**0.31**	Strong	Ideal strong pattern
**Fig. 4j**	16.77	5.12	5.12	0.61	**39.31**	**10.32**	**10.27**	**0.52**	Moderate	{100} and {002} arc intensities very strong
**Fig. 4k**	16.64	3.05	3.05	0.37	**38.97**	**8.23**	**8.28**	**0.42**	Strong	{100} arc intensity weak
**Fig. 4l**	16.64	4.35	4.35	0.52	**38.97**	**5.95**	**5.95**	**0.31**	Strong	{100} and {002} arc intensities relatively strong
